# Newcastle Disease virus infection activates PI3K/Akt/mTOR and p38 MAPK/Mnk1 pathways to benefit viral mRNA translation via interaction of the viral NP protein and host eIF4E

**DOI:** 10.1371/journal.ppat.1008610

**Published:** 2020-06-30

**Authors:** Yuan Zhan, Shengqing Yu, Shen Yang, Xusheng Qiu, Chunchun Meng, Lei Tan, Cuiping Song, Ying Liao, Weiwei Liu, Yingjie Sun, Chan Ding

**Affiliations:** 1 Shanghai Veterinary Research Institute, Chinese Academy of Agricultural Science, Shanghai, P.R. China; 2 Jiangsu Co-innovation Center for Prevention and Control of Important Animal Infectious Diseases and Zoonoses, Yangzhou University, Yangzhou, P.R. China; University of Georgia, UNITED STATES

## Abstract

Newcastle disease virus (NDV), a member of the *Paramyxoviridae* family, can activate PKR/eIF2α signaling cascade to shutoff host and facilitate viral mRNA translation during infection, however, the mechanism remains unclear. In this study, we revealed that NDV infection up-regulated host cap-dependent translation machinery by activating PI3K/Akt/mTOR and p38 MAPK/Mnk1 pathways. In addition, NDV infection induced p38 MAPK/Mnk1 signaling participated 4E-BP1 hyperphosphorylation for efficient viral protein synthesis when mTOR signaling is inhibited. Furthermore, NDV NP protein was found to be important for selective cap-dependent translation of viral mRNAs through binding to eIF4E during NDV infection. Taken together, NDV infection activated multiple signaling pathways for selective viral protein synthesis in infected cells, via interaction between viral NP protein and host translation machinery. Our results may help to design novel targets for therapeutic intervention against NDV infection and to understand the NDV anti-oncolytic mechanism.

## Author summary

Viruses are obligate intracellular parasites and have no protein translation machinry of their own. Therefore, viruses remain exclusively dependent on host translation machinery to ensure viral protein synthesis and progeny virion production during infection. We previous reported that Newcastle disease virus (NDV) shutoff host and facilitate viral mRNA translation by activating PKR/eIF2α signaling cascade. Here, we demonstrated that NDV infection up-regulated host cap-dependent translation machinery by activating PI3K/Akt/mTOR and p38 MAPK/Mnk1 pathways. Furthermore, NDV NP protein was found to be important for selective cap-dependent translation of viral mRNAs. Our findings highlight a new strategy how virus used host translation machinery for selective viral protein synthesis.

## Introduction

Viruses remain exclusively dependent on the translation machinery of their host to ensure viral protein synthesis and progeny virion production during infection. Viral strategies to dominate the host translation machinery target almost all the steps of eukaryotic translation. Translation initiation is considered to be a rate limiting process for eukyarotic protein synthesis, which is achieved by a cap-dependent scanning mechanism through sequential formation of several initiation complexes [1]. Cap-dependent translation begins with the recognition of eukaryotic mRNAs 7-methyl GTP cap by eukaryotic translation initiaton factor 4F (eIF4F), which is composed of eIF4E, eIF4A and eIF4G [1–3]. eIF4E binding to the 5' cap structure is the first step of cap-dependent translation initiation; eIF4A is an RNA helicase that unwinds secondary structure in the 5'-UTR of mRNAs and facilitates scanning by the 40S ribosomal subunit towards the initiation codon; eIF4G promotes assembly of eIF4E and eIF4A into the eIF4F complex, and functions as a scaffold protein by linking the mRNA and the small ribosomal subunit by means of multiple protein-protein interactions [4–6]. eIF4F function is regulated by phosphorylation signaling cascades, the ability of eIF4E to functionally associate with eIF4G to assemble eIF4F complex is a major target for regulationg translation initiation [7, 8]. The activity of eIF4E is inhibited by the unphosphorylated isoform of eIF4E binding proteins (4E-BPs) [9]. In its hypophosphorylated form, 4E-BPs sequesters eIF4E and blocks the binding site for eIF4G, inhibiting cap-dependent translation. In contrast, hyperphosphorylation of the 4E-BPs by the mammalian target of rapamycin (mTOR) results in the release of eIF4E, allowing eIF4G to bind eIF4E to assemble an active eIF4F complex and stimulate cap-dependent translation [10, 11]. mTOR is a downstream kinase within the PI3k/Akt/mTOR signaling pathway. In addition to hyperphosphorylation of 4E-BP1, mTOR also regulates translation via the activation of p70S6K and the subsequent phosphorylation of rpS6 correlates with an increase in translation of mRNA transcripts that contain an oligopyrimidine tract in their 5' untranslated regions [12]. Another pathway that regulates translation initiation is p38 MAP kinase-interacting serine/threonine-protein kinase 1 (Mnk1), which catalyzes eIF4E Ser209 phosphorylation [13]. Mnk1 can be activated by either the Erk or p38 kinases at Thr197 and Thr202 which are essential for Mnk1 kinase activity [14, 15]. Mnk1 is also associated with the eIF4F complex via its interaction with the C-terminal region of eIF4G and its recruitment to eIF4G is critical for eIF4E phosphorylation [16]. Although the consequence of eIF4E phosphorylation is still questionable, it has been shown to control specific mRNA translation and associate with increased translation rates [17]. In addition to eIF4F, eIF2α is a a negative regulator for eukaryotic translation initiation. Phosphorylation of eIF2α at Ser 51 impairs the exchange of GDP for GTP mediated by eIF2B in the ternary complex eIF2-GTP-tRNA^**Met**^. Since eIF2B is present in limiting quantities, slight changes in eIF2α phosphorylation can result in the drastic suppression of global protein synthesis [18].

Given the essential role of eIF4F in cellular mRNA translation, many viruses target eIF4F during infection to facilitate their own replication by distinct strategies. To compete with the host mRNAs for translation factors and ribosomes, many viruses induce the shutoff of global cellular protein synthesis. For instance, enteroviruses, retroviruses and caliciviruses can encode proteases that cleave eIF4G to suppress cap-dependent translation [19]. Other viruses such as vesicular stomatitis virus, polipvirus and simian vacuolating virus 40 induce the dephosphorylation of 4E-BP1 to arrest eIF4E and limit eIF4F formation [20]. Inhibiting host cap-dependent translation does not prevent viral mRNA translation, as many RNA viruses contain an internal ribosome entry site (IRES) in the 5’-untranslated region that direct cap-independent translation. By contrast, many DNA viruses rely on cap-dependent translation and stimulate eIF4F activity. Herpesvirues, human cytomegalomvirus, Epstein-Barr virus, vaccinia virus and asfarvirus all activate cap-dependent mechanism, promoting mTOR and eIF4E phosphorylation, 4E-BP1 hyperphosphorylation, as well as eIF4F assembly [20]. Many such DNA viruses even compartmentalize and concentrate the translational apparatus within viral replication compartments to facilitate viral protein production. Moreover, because of the essential role of eIF2α in translation initiation, viruses have also developed various mechanisms to regulate eIF2α phosphorylation.

Newcastle disease virus (NDV), a member of the *Paramyxoviridae* family, is a single-stranded, nonsegmented, negative-sense RNA virus with a natural avian host range. NDV is also known to be an oncolytic virus, which can selectively replicate in tumor cells to induce apoptosis in late stages of infection [21] and trigger the formation of autophagosomes in U251 glioma cells to enhance virus replication [22]. The NDV genome contains six major genes that encode the nucleocapsid protein (NP), phosphoprotein (P), matrix protein (M), fusion protein (F), hemagglutinin-neuraminidase (HN), and large polymerase protein (L) in the order of 3'-NP-P-M-F-HN-L-5'. For virus genome replication, NP protein binds to the viral genome and forms a ribonucleoprotein complex with P and L proteins, acting as a template for viral genome replication and transcription [23]. NDV mRNAs are capped and polyadenylated by the L protein during synthesis, and then translated by cellular translational machinery [24]. Increasing evidence suggest that signaling pathways related to the regulation of eIF4F function, such as PI3K/Akt/mTOR, Raf/MEK/Erk, and p38 MAP kinase are involved in discrete steps of the NDV viral life cycle [22, 25, 26]. Our previous studies indicated that Akt, mTOR and p70S6K phosphorylation is beneficial to NDV replication by inducing autophagy [26, 27]. However, the regulation mechanism of translational machinery in NDV-infected cells and the detailed cellular pathways have not been elucidated. The aim of the current study was to investigate how NDV infection mainpulates the host translational machinery to facilitate the viral mRNA translation. We found that NDV infection enhanced eIF4F complex assembly and thereby cellular translational machinery activation by both PI3K/Akt/mTOR and p38 MAPK/Mnk1 pathways, through binding NDV NP protein to eIF4E to facilitate the selective translation of viral mRNAs. Our findings provide important insight of how this virus manipulates infected host cells to promote its life cycle which is helpful for design novel targets for therapeutic intervention against NDV infection and understanding of the NDV anti-oncolytic mechanism.

## Materials and methods

### Cell culture and virus

The human epithelial carcinoma cell line HeLa, the human emborynic kidney (HEK) 293T cell line and the chicken fibroblast DF-1 cell line were purchased from the American Type Culture Collection (ATCC) and maintained in Dulbecco's modified Eagle's medium (DMEM) supplemented with 10% fetal calf serum (Thermo Fisher Scientific, Waltham, MA, USA), in an incubator at 37°C containing 5% CO_**2**_. NDV strain Herts/33 was obtained from the China Institute of Veterinary Drug Control (Beijing, China). To inactivate NDV Herts/33 strain with UV-irradiation, the virus stocks were dispersed in a 10 cm tissue culture dish, and placed under a UV lamp (20 W) for 30 min. The absence of virus infectivity after UV treatment was confirmed by the lack of replication in 9-day-old SPF embryonated chicken eggs.

### Antibodies and reagents

CGP57380 (C0993) was purchased from Sigma-Aldrich and used at 20 μM. Rapamycin (#9904), LY294002 (#9901), U0126 (#9903) and SB203580 (#5633) were purchased from Cell Signaling Technology (Danvers, MA, USA) and used at 100 nM, 20 μM, 10 μM, and 10 μM respectively. Mouse monoclonal antibodies against NDV phosphoprotein (P protein) and nucleocapsid protein (NP protein) were prepared in our laboratory. Puromycin and mouse monoclonal anti-puromycin 12D10 (MABE343) were purchased from Merck Millipore (Billerica, MA, USA). Mouse monoclonal anti-FLAG M2 antibody (F1804) and anti-β-actin antibody (A1978) were purchased from Sigma-Aldrich (St. Louis, MO, USA), and other primary antibodies were from Cell Signaling Technology. Horseradish peroxidase (HRP)-conjugated goat anti-rabbit or -mouse secondary antibody, tetramethyl rhodamine isothiocyanate (TRITC)-conjugated goat anti-mouse and fluorescein (FITC)-conjugated goat anti-rabbit secondary antibody were purchased from Jackson ImmunoResearch Laboratories (West Grove, PA, USA). Small interfering RNAs (siRNAs) designed specfically for human Mnk1 (sc-39106), eIF4E (sc-35284) and eIF4G (sc-35286) were from Santa Cruz Biotechnology (Dallas, TX, USA). RC DC Protein Assay kit (500–0199) was purchased from Bio-Rad.

### Plasmid construction and transfection

To construct plasmids expressing Flag-tagged eIF4E and eIF4G, total RNA of HeLa cells was extracted and reverse transcribed. eIF4E and eIF4G fragments were PCR amplified using specific primer pairs and cloned into the NotI/XhoI or EcoRI/XhoI sites of p3×FLAG-CMV-14 vector (Sigma) to generate the p3×FLAG-eIF4E and p3×FLAG-eIF4G plasmid respectively. The NDV NP cDNA (GenBank accession no. AY741404.1) was cloned into vector p3×FLAG-CMV-14 (Sigma) and pCMV-HA (Clontech) to generate the p3×FLAG-NP (with a Flag tag) and pCMV-HA-NP (with a HA tag) plasmids repectively. For prokaryotic expression of the GST-tagged NP protein, the NDV NP cDNA was cloned into the pGEX-6p-1 vector (GE Healthcare) to generate the pGEX-NP. Primers used in this study are listed in S1 Table. All plasmids were verified by sequencing. For transfection, cells were seeded in six-well plates and transfected at 70% confluency with corresponding plasmids DNA (2 μg each) by FuGENE HD transfection reagent (E2312, Promega) according to the manufacturer's instructions.

### Virus infection and inhibitor treatment

HeLa cells were infected with NDV Herts/33 at a multiplicity of infection (MOI) of 5, or mock-treated with the same medium without virus at 37°C. Following a 1-h adsorption period, unattached viruses were removed and cells were washed with PBS and then cultured in fresh medium. Viral titers were determined on DF-1 cells as median tissue culture infective dose (TCID_**50**_) calculated using the Reed-Muench formula [28]. Viral titers were expressed as percentage of control. Triplicate wells were tested for each experiment, and the full experiment was repeated twice. Virus replication was determined by quantifying NDV P and NP protein expression, using Western blot as described previously [29].

The optimal concentrations of drugs used in this study were determined by WST-1 assay according to the manufacturer’s guidelines (Beyotime Biotechnology, Shanghai, China) (S1 Fig). For inhibitor experiments, the 1,000× stock of inhibitors were prepared in DMSO. DMEM containing DMSO was used for the mock-treatment. Cells were pretreated with the inhibitor for 1 h, then infected with the virus for 1 h, washed with PBS for three times, and placed in serum-free media containing fresh inhibitor.

### Confocal fluorescence microscopy

Fixation, permeabilization, and confocal microscopy observation were performed as described previously [30]. The primary monoclonal anti-eIF4E and anti-NDV NP antibodies were used at 1:100 and at 1:500 dilution respectively. The localization of eIF4E and NP protein was visualized using a Nikon C1-si confocal fluorescence microscope (Nikon Instruments, Inc.).

### Western blot

HeLa cells infected with NDV or mock-treated were washed with PBS and lysed with 300 μL lysis buffer at 4°C at 2, 4, 6, 8, 12 and 24 h post infection (hpi) as described previously [31]. The lysates were cleared by centrifugation for 15 min at 12,000 ×*g*. The resulting supernatants were subjected to SDS-PAGE under reducing conditions, and transferred onto nitrocellulose membranes (Whatman International). Membranes were blocked for 1 h at room temperature with non-fat milk solution (5% in Tris-buffered saline) containing 0.1% Tween 20, then reacted with primary antibodies overnight at 4°C and HRP-conjugated secondary antibodies for 1 h at room temperature. After extensive washing, protein bands were detected using Supersignal west pico or femto chemiluminescence kit (Thermo Fisher Scientific). Protein abundance was quantified by densitometric scanning using the Quantity One 1-D software (version 4.4.0) (Bio-Rad Laboratories).

### ^35^S metabolic labeling

Synthesis of cellular and NDV viral proteins was analyzed by ^**35**^S metabolic labeling. HeLa cells were infected with NDV or mock-treated, then labeled with 100 μCi ^**35**^S Express (Perkin Elmer) at different time points in methionine- and cystein-free DMEM (Gibco) at 37°C for 1 h. Cells were lysed and subjected to SDS-PAGE, ^**35**^S-labeled proteins were visualized and quantitated with a phosphorimaging plate in Typhoon FLA 9000 (GE).

### Ribopuromycilation assay

Ribopuromycilation assay, the nonradioactive method, was performed as previously to monitor the protein synthesis of cells infected with NDV [32, 33].

### RNA interference

siRNAs targeting eIF4E and eIF4G were performed at a final concentration of 100 nM and 50 nM respectively. HeLa cells grown to 60 to 70% confluence in 6-well plates were transfected with siRNAs with Lipofectamine 2000 (Invitrogen) as described previously [26]. Western blot was used to analyze eIF4E and eIF4G production with specific primary antibodies. For Mnk1 knockdown, siRNA against Mnk1 was used at a final concentration of 50 nM. HeLa cells grown to 60 to 70% confluency were transfected and/or treated with 100 nM Rapamycin. Cells were collected at the indicated time points and subjected to Western blot analysis.

### 7-methyl GTP Sepharose 4B pull-down assay

eIF4F complexes were pulled down from protein lysates using 7-methyl GTP sepharose 4B beads (GE Healthcare). HeLa cells were infected with NDV, or transfected with the p3×FLAG-NP plasmid, then harvested and lysed at different time points. Protein concentration was determined using the Bio-Rad DC protein assay system (Bio-Rad). eIF4F complexes were immunoprecipitated from 1 mg of total protein by incubating with 50 μl of 7-methyl GTP Sepharose 4B beads in a total volume of 500 μl of lysis buffer. After 8 h of incubation at 4°C, the beads were washed twice in lysis buffer. The washed beads were then boiled in 2× SDS protein loading buffer and the proteins were subjected to SDS-PAGE followed by Western blot as described. Densitometric values of the bands were normalized against β-actin bands from the lysates.

### Immunoprecipitation

For immunoprecipitation, HeLa cells were infected with NDV or transfected with the p3×FLAG-NP plasmid. To perform co-immunoprecipitation, 293T cells were co-transfected with 3×FLAG tagged eIF4E or eIF4G, and HA-tagged NP plasmids. Cells were harvested, washed and lysed in NP-40 lysis buffer (50 mM Tris pH 8.0, 150 mM NaCl, 0.5% NP-40, 0.5 mM EDTA) containing 1 mM phenylmethylsulfonyl fluoride (PMSF) and 1 mg/mL protease inhibitor cocktail (Roche), then centrifuged at 12,000 ×*g* for 15 min. After protein quantification, 1 mg of total protein was incubated overnight with specific primary antibodies and the complexes were pulled down by incubation with protein-G agarose beads (Life Technologies) for 2 h. The beads were washed twice with NP-40 lysis buffer, resuspended in 2× SDS protein loading buffer, boiled, and subjected to SDS-PAGE. Immunoprecipitated proteins were detected by Western blot analysis. Run-off lysates were used for Western blot to detect β-actin for sample normalization.

### GST pull-down assay

NDV NP subcloned into pGEX-6p-1 vector was transformed and expressed in *Escherichia coli* BL21 (DE3) strain. GST-NP fusion protein was purified using glutathione-Sepharose beads (GE Healthcare), according to the manufacturer’s instructions. The GST Pull-down assays were carried out as described [34]. In brief, GST or GST-NP cells was conjugated to glutathione beads and incubated with 293T cell lysates overexpressing FLAG-eIF4E for 2 h at 4°C. After washing to remove the unbound molecules, the protein complexes were eluted with loading buffer, and detected by Western blot assay using anti-GST and anti-FLAG antibodies.

### Polysome profile analysis

HeLa cells were infected with NDV or mock-treated for 8 h, then incubated with 100 μg/mL cycloheximide (CHX) for 15 min at 37°C. Cells were washed and lysed for polysome fractionation by sucrose density gradient ultracentrifugation according to previous reports [35, 36]. For analysis of dissociated polysomes, cycloheximide was omitted from the polysome lysis buffer and replaced with 20 mM EDTA. The lysates were clarified by centrifugation at 12,000 ×*g* for 15 min at 4°C, and supernatant was resolved on a linear sucrose gradient (7–47% in buffer containing 20 mM Tris-Cl, pH 8.0, 140 mM KCl, 1.5 mM MgCl_**2**_, 1 mM DTT, 1 mg/mL heparin) by centrifugation at 40,000 ×*g* at 4°C for 3 h. After centrifugation, each 1-mL fraction was collected and analyzed by UV absorbance at 254 nm. For analysis of proteins in polysomes, total proteins from each sucrose gradient fraction were precipitated with trichloroacetic acid (TCA) and analyzed by Western blot analysis. To examine the distribution of ribosomal RNA in the gradients, total RNA was extracted with phenol/chloroform, precipitated with ethanol, and resuspended in dH_**2**_O for quantitative RT-PCR analysis. Absolute quantitative real-time PCR-based analysis of NDV NP mRNA was performed as previously [37]. Primer sequences are listed in S1 Table.

### Translation inhibition assay

Stable expression of NDV NP protein in HeLa cells was achieved using lentiviral expression system containing pWPXL (12257), pMD2.G (12259) and psPAX2 (12260) plasmids purchased from Addgene (Cambridge, MA, USA). The recombinant pWPXL-NP plasmid was constructed by inserting the NDV NP cDNA into the BamHI and NheI sites of the pWPXL vector. The lentiviral vector coding for eGFP (pWPXL-empty) was used as the control. The pWPXL-NP and pWPXL-empty lentiviruses were prepared in 293T cells and titered as previously described [38], then used to transduce HeLa cells in the presence of 8 μg/mL polybrene (Sigma). The expression of NDV NP protein was confirmed by Western blot. HeLa-NP cells and HeLa-eGFP cells were plated in 24-well plates, the pRL-TK vector encoding a Renilla luciferase reporter gene was transfected and incubated for 24 h. The luciferase activity was analyzed with the Renilla Luciferase Assay System (Promega) and measured with a Glomax-multi detection system (Promega). Meanwhile, the total RNA was analyzed for Renilla luciferase transcripts using quantitative RT-PCR analysis. Primer sequences are listed in S1 Table.

### Statistical analysis

The band intensities of Western blot were calculated using ImageJ software (NIH). Statistical significance was determined by two-tailed Student *t*-test or one-way analysis of variance (ANOVA) test where applicable, using GraphPad Prism 5.0 software (GraphPad Software). Data were considered statistically significant at p < 0.05. Values were expressed as mean ± standard error of the mean.

## Results

### NDV infection shut off host protein for viral protein synthesis and activated cap-dependent translation factors

**Fig 1:**
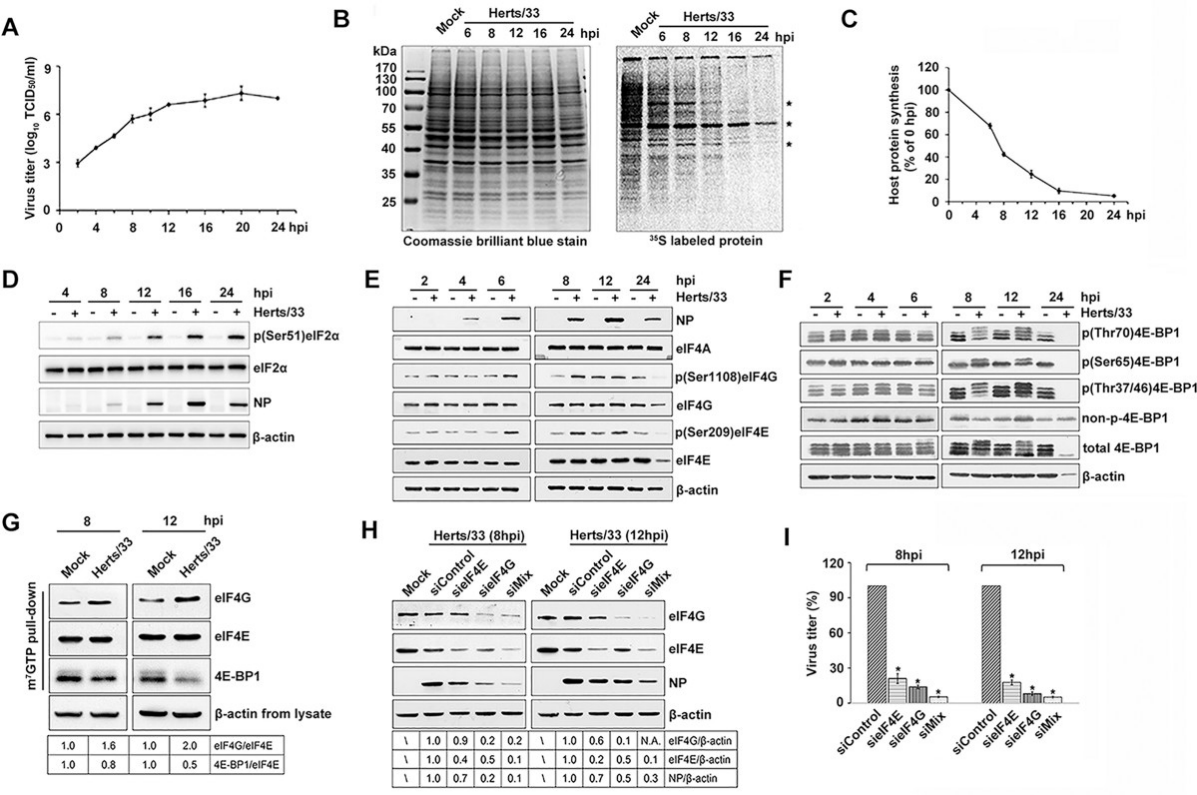
NDV infection induces the inhibition of host protein synthesis and eIF4F complex phosphorylation and assembly. (A) The growth curve of NDV in HeLa cells. HeLa cells were infected with 5 MOI of Herts/33. Supernatants were harvested at indicated times and were subjected to TCID_50_ assay. TCID_50_ was calculated using Reed-Munch mathematical analysis. (B) HeLa cells infected with Herts/33 were labeled with 100 μCi of [^35^S] methionine/cysteine for 1 h and collected at the indicated time. Labeled proteins were analyzed by SDS-PAGE followed by fluorography and autoradiography. Asterisks (*) indicate newly synthesized proteins detected only in Herts/33 infected cells. Molecular weight standards appear in the leftmost lane and their sizes (kDa) are indicated in the margin. Coomassie brilliant blue staining of the autoradiograph gel were performed to confirm the equivalence of protein loading. (C) Quantitation of host protein synthesis in NDV-infected HeLa cells. The rates of protein synthesis were determined as fold changes of host protein synthesis in NDV-infected cells compared to that in mock-infected cells. (D) HeLa cells were mock-infected or infected with NDV, and harvested at indicated times. Total protein was isolated, and equivalent amounts were fractionated by SDS-PAGE, and analyzed by immunoblotting using antibodies recognizing phospho-eIF2α (p-eIF2α), total eIF2α, NP, and β-actin. (E) Phosphorylation of eIF4E and eIF4G is stimulated in NDV infected HeLa cells. HeLa cells were either mock-infected (Mock) or infected with NDV (5MOI). At the indicated times points, total protein was isolated, and equivalent amounts were fractionated by SDS-PAGE, and analyzed by immunoblotting using antibodies recognizing phospho-eIF4E (p-eIF4E), total eIF4E, phospho-eIF4G (p-eIF4G), total eIF4G, eIF4A, NP and β-action. (F) The phosphorylation status of 4E-BP1 was analyzed using antibodies against total 4E-BP1, phopho-4E-BP1 (Thr70), phopho-4E-BP1 (Ser65), phopho-4E-BP1 (Thr37/46) and non-phospho-4E-BP1 (Thr46). (G) eIF4F complex were precipitated from the lysates mentioned in (D) by m7GTP pull down assay. The precipitates were boiled in 2×loading buffer and the component proteins were analyzed by western blotting as shown. The band intensities of eIF4G and 4E-BP1 were determined and normalized to eIF4E. The intensity bind ratio for mock was given a value of 1 and indicated in the frames below the blots. (H) HeLa cells were transfected with siControl, sieIF4E, sieIF4G or a mixture of sieIF4E and sieIF4G (siMix) as indicated. After 48 h, cells were infected with 5 MOI of NDV and samples were recovered after 8 and 12 hpi respectively. Depletion of eIFs was examined by Western blotting against eIF4G and eIF4E. Accumulation of viral proteins was analyzed using specific antibodies against NP protein. The band intensities of eIF4G, eIF4E and NP were determined and normalized to β-actin. The intensity bind ratio for cells transfected with nontarget siRNA (siControl) was given a value of 1 and indicated in the frames below the blots. (I) Supernatants from transfected cells were collected at the indicated times and determined by TCID_50_ assay. Viral titers were expressed as percentage of control. Data are presented as means from three independent experiments. Significance was analyzed by one-way analysis of variance followed by Dunnett’s test (*, p<0.05; ns, not significant).

After infection of HeLa cells, NDV titers increased with time progression and peaked at 20 hpi at approximately 7.57 log_10_ TCID_50_/mL, on the contrary, the host protein synthesis decresed accordingly (Fig 1A, 1B and 1C). Radio labeling experiments showed the apparent bands in NDV-infected cells (marked by asterisks in Fig 1B and S3B Fig), which were probably viral proteins according to previous reports [39, 40]. The results showed that viral proteins became the predominant translation products at 8 hpi, while host *de novo* protein synthesis was gradually decreased until 24 hpi (Fig 1B and 1C). Western blot further indicated an increased NDV NP protein and eIF2α phosphorylation from 8 through 24 hpi (Fig 1D). The results of nonradioactive ribopuromycilation assay further confirmed the inhibition of global protein synthesis in NDV-infected cells (S2 Fig). Similar pattern of protein synthesis were also observed in chicken-derived DF-1 cells infected with NDV (S3 Fig). These results suggested that NDV infection led to shutoff of cellular protein for viral protein translation and eIF2α activation.

To determine whether NDV infection functions on eIF4F, we examined eIF4G and eIF4E phosphorylation of the infected cells by Western blot. The results indicated an increased phosphorylation of eIF4G at 6 and 8 hpi, and of eIF4E at 6, 8 and 12 hpi, correlating with robust synthesis of viral protein (Fig 1E). Treating HeLa cells with UV-inactivated NDV Herts/33 did not alter eIF4F phosphorylation (S4 Fig), indicating that the upregulation of eIF4G and eIF4E phosphorylation was induced by NDV infection. 4E-BP1 acts as an inhibitor of cap-dependent translation, as it competes with eIF4G for eIF4E binding. We further examined 4E-BP1 phosphorylation, and detected increased 4E-BP1 phosphorylation at 8 and 12 hpi on three amino acids examined in NDV-infected cells as compared with the control cells (Fig 1F), suggesting that in addition to inducing eIF4G and eIF4E phosphorylation, NDV infection also results in hyperphosphorylation of 4E-BP1 to activate cap-dependent translation apparatus.

Next, eIF4F complexes were precipitated using an m^7^GTP pull-down assay from NDV-infected and mock-treated cells to investigate the binding of eIF4G and eIF4E by Western blot. We found that eIF4G was incresed and 4E-BP1 was decresed in the eIF4F complex as compared with mock-treated cells, suggesting that NDV infection promotes the assembly of the active eIF4F complex (Fig 1G). Moreover, Western blot showed that NDV NP protein was significantly reduced in HeLa cells with 80–90% deletion of eIF4G, eIF4E or both eIF4E/eIF4G using siRNAs, as compared to control cells (Fig 1H), especially when both eIF4E and eIF4G were depleted. The effect of eIF4F depletion on NDV production was also investigated, indicating the produced progeny virus was severely reduced at 8 and 12 hpi (Fig 1I). Collectively, these results suggest that NDV infection shut off host protein translation but meanwhile activates cap-dependent translation.

### PI3K/Akt/mTOR pathway is required for eIF4F assembly and viral protein synthesis during NDV infection

**Fig 2:**
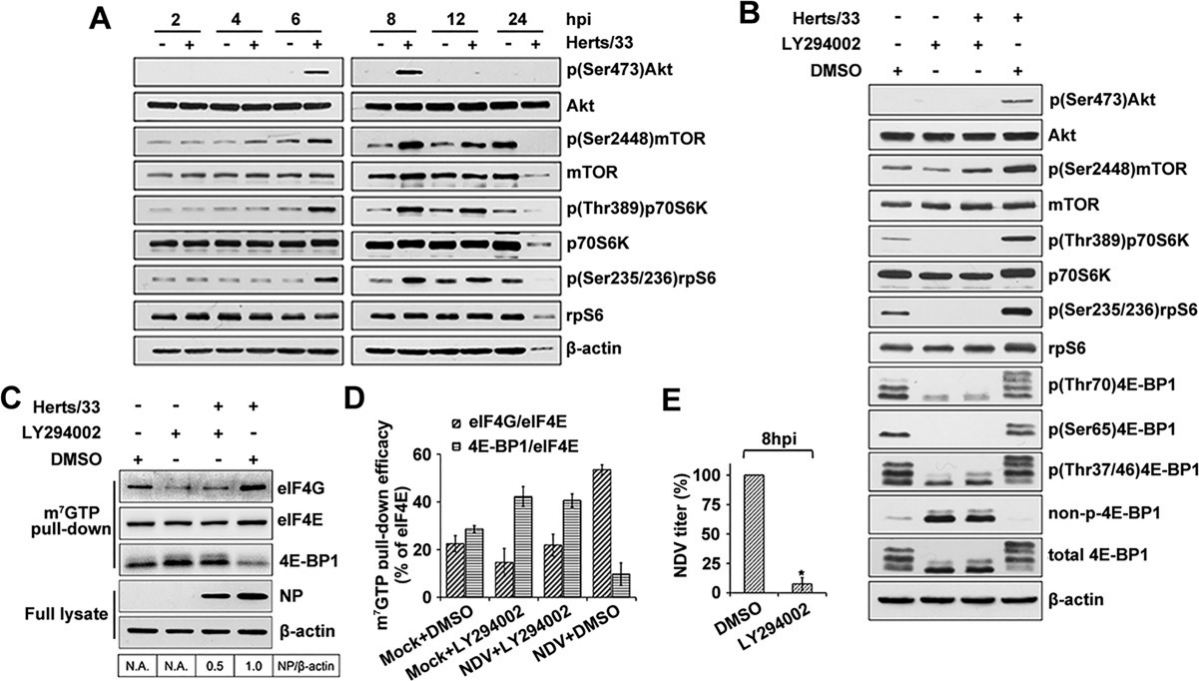
NDV activates PI3K/Akt/mTOR pathway to phosphorylate downstream proteins that regulate translation in order to promote the eIF4F assembly and the production of NDV virus proteins. (A) HeLa cells infected with 5MOI of NDV Herts/33 were harvested at the indicated times, both phosphorylation and total of Akt, mTOR, p70S6K and rpS6 were analyzed by western blotting. (B) PI3K specific inhibitor LY294002 suppressed Akt/mTOR/4E-BP1 phosphorylation induced by the virus infection. Cells preincubated with medium in the absence of LY294002 and then mock infected or infected with the virus were used as the negative and positive control, respectively. HeLa cells were pretreated with LY294002 and infected with NDV at an MOI of 5 in the presence of the inhibitor, and then processed for Western blotting at 8hpi with antibodies as shown. (C) eIF4G-eIF4E association and NDV protein synthesis are abrogated by LY294002 treatment. HeLa cells were pretreated with 20 μM LY294002. After 2 h cells were infected with NDV (5 MOI) in the continuous presence of the compounds. At 8 hpi cells were incubated with 7-Methyl GTP-Sepharose 4B beads. eIF4G, eIF4E, 4E-BP1, NP and β-actin were detected in eluted or full lysate by Western blotting. The band intensities of NP was determined and normalized to β-actin. The intensity bind ratio for infected cells in the absence of LY294002 was given a value of 1 and indicated in the frames below the blots. (D) The efficacy of cap Sepharose pull-down in the presence of LY294002 was determined using immunoblotting for eIF4E, eIF4G and 4E-BP1. The efficacies of eIF4G and 4E-BP1 pull-down are expressed and normalized as the percentage of the eIF4E. (E) The extracellular virus yields were determined by TCID_50_ assay at 8 hpi and expressed as percentage of control. Data are presented as means from three independent experiments. Significance is analyzed by two-tailed Student's t-test (*, p<0.05; ns, not significant).

To determine the upstream intracellular pathways involved in translation regulation by NDV infection, the PI3K/Akt/mTOR pathway were first examined [35, 41]. The results showed that phosphorylation of Akt and mTOR, as well as two downsteam effectors p70S6K and rpS6 was all enhanced at 6 and 8 hpi (Fig 2A), suggesting the Akt/mTOR signaling pathway is activated during NDV infection. In view of the fact that Akt can be activated by either PI3K-dependent or -independent ways [41, 42], we next analyzed the role of PI3K in Akt/mTOR signaling pathway following NDV infection. LY294002, a potent and specific inhibitor of PI3K [43] was used for the inhibition assay. The result showed that LY294002 inhibited phosphorylation of Akt, mTOR, p70S6K, rpS6, and 4E-BP1 (Fig 2B), suggesting a downstream inhibition of mTOR activity by PI3K inhibitor. These results indicate that the virus inducing Akt/mTOR activation was mediated by PI3K.

The effect of inhibitor LY294002 on the formation of eIF4F complexes and viral protein synthesis in NDV-infected cells was then examined. HeLa cells were mock-treated or infected with NDV in the presence of LY294002 or a DMSO solvent control. At 8 hpi, cell lysates were prepared and eIF4F was recovered by m^7^GTP pull-down. Inhibition of upstream PI3K signaling with LY294002 resulted in a reduction of eIF4E-eIF4G, an increase of eIF4E-bound 4E-BP1, and a 50% decrease in viral protein production in the NDV infected HeLa cells (Fig 2C and 2D). In addition, NDV titer was 10-fold reduced by LY294002 treatment when measured at 8 hpi (Fig 2E). The results demonstrated that the inhibition of PI3K signaling increases 4E-BP1 quantities and subsequently prevents eIF4F assembly, and therefore leads to the inhibition of viral mRNA translation in NDV-infected cells. However, it should be noted that the translation of viral mRNA was not completely blocked by LY294002, suggesting virus may use another arm of the signaling pathway to achieve protein translation.

### NDV infection induced an mTOR kinase-dependent eIF4G phosphorylation but not rapamycin-sensitive 4E-BP1 phosphorylation

**Fig 3:**
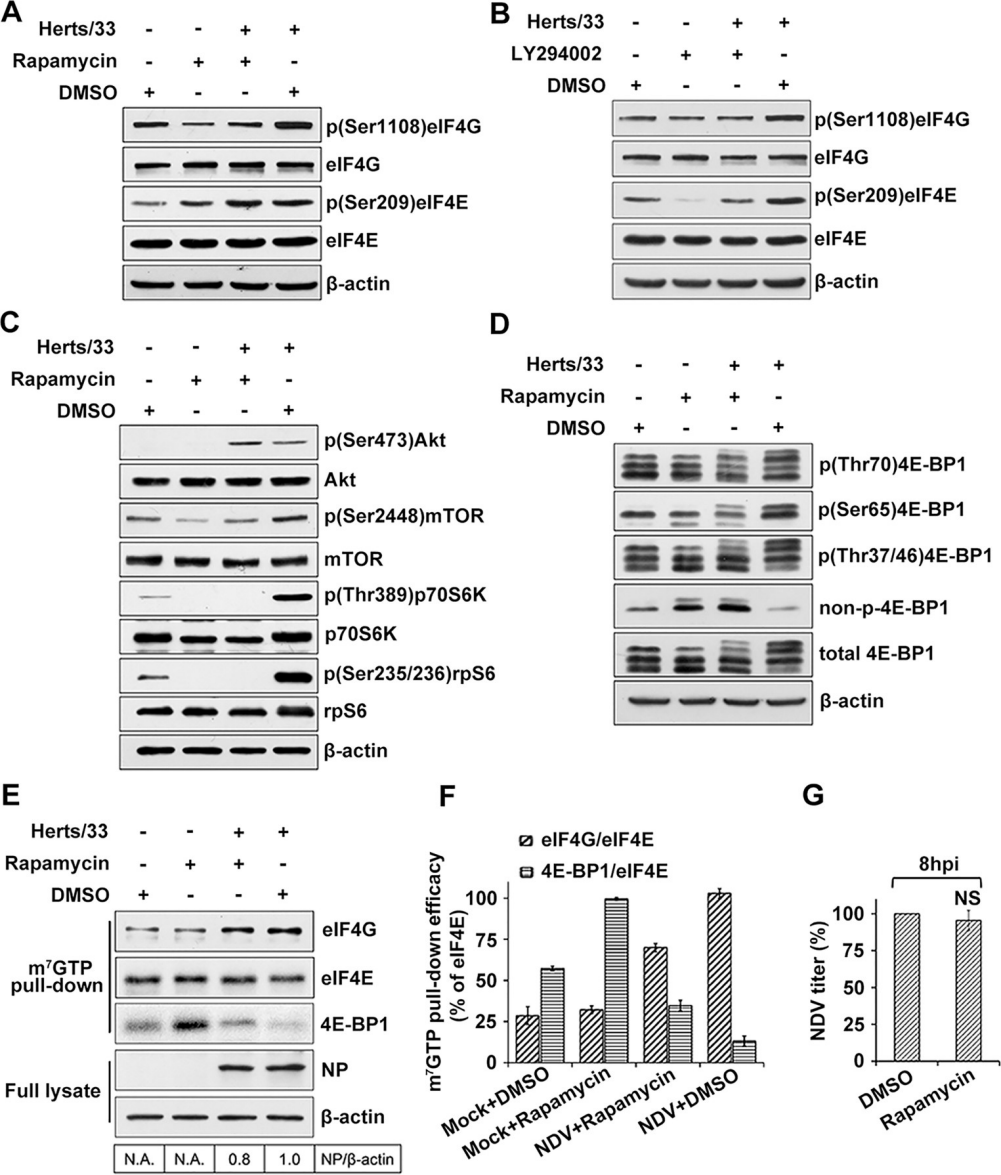
Phosphorylation of eIF4G requires mTOR activity and NDV mediated 4EBP1 phosphorylation is rapamycin resistant. (A-B) NDV infection increases level of phosphorylated eIF4G through mTOR activation. HeLa cells were non-treated or pretreated for 2 h with rapamycin 100 nM (A) or LY294002 20 μM (B) then infected with 5MOI of NDV. Cells were then lysed with buffer sample at 8 hpi. Amounts of phospho-eIF4G and total eIF4G were analyzed by Western blotting. β-actin was detected as a load control. (C) Effect of NDV infection on total levels and phosphorylation status of mTOR effectors in the presence and absence of rapamycin. HeLa cells were pretreated 2h with 100nM rapamycin and infected or mock infected in the presence with rapamycin for 8h after which the cells were harvested, protein lysates prepared and phosphorylation of mentioned molecules were subsequently analyzed by immunobloting. (D) NDV induced 4E-BP1 phosphorylation is rapamycin insensitive. As in C, the phosphorylation status of 4E-BP1 was analyzed using antibodies against total 4E-BP1, phopho-4E-BP1 (Thr70), phopho-4E-BP1 (Ser65), phopho-4E-BP1 (Thr37/46) and non-phospho-4E-BP1 (Thr46). (E) Rapamycin treatment can’t suppress the eIF4F assembly and production of NDV virus proteins. HeLa cells were pretreated with 100 nM Rapamycin. After 2 h cells were infected with NDV (5 MOI) in the continuous presence of the compounds. At 8 hpi cells were incubated with 7-Methyl GTP-Sepharose 4B beads. eIF4G, eIF4E, 4E-BP1, NP and β-actin were detected in eluted or full lysate by Western blotting. The band intensities of NP was determined and normalized to β-actin. The intensity bind ratio for infected cells in the absence of Rapamycin was given a value of 1 and indicated in the frames below the blots. (F) The efficacy of cap Sepharose pull-down in the presence of Rapamycin was determined using immunoblotting for eIF4E, eIF4G and 4E-BP1. The efficacies of eIF4G and 4E-BP1 pull-down are expressed and normalized as the percentage of the eIF4E. (G) The extracellular virus yields were determined by TCID50 assay at 8 hpi and expressed as percentage of control. Data are presented as means from three independent experiments. Significance is analyzed by two-tailed Student's t-test (*, p<0.05; ns, not significant).

To investigate the participation of mTOR in the NDV-induced phosphorylation of eIF4G, HeLa cells were pre-incubated with rapamycin or LY294002 for 2 h before NDV infection. Treatment with rapamycin suppressed eIF4G phosphorylation in both NDV-infected and mock-treated cells, however, eIF4E phosphorylation was not affected (Fig 3A). When treated with LY294002, both eIF4G and eIF4E phosphorylation were suppressed in NDV-infected and mock-treated cells, and the suppression was more severe for eIF4E than eIF4G. (Fig 3B). The low level of eIF4E phosphorylation was likely due to the collapse of eIF4F complex, which prevents proper positioning of the eIF4G bound kinase Mnk1 in proximity to its substrate, eIF4E [16, 44, 45]. These data suggested mTOR activity is necessary for inducing eIF4G phosphorylation post NDV infection. To investigate the effect of mTOR inhibition on NDV induced 4E-BP1 hyperphosphorylation, treatment with rapamycin was performed. Rapamycin treatment inhibited mTOR, p70S6K and rpS6 phosphorylation in NDV-infected cells. Among them, p70S6K and rpS6 phosphorylation was completely blocked. In contrast, the phosphorylation of the upstream target Akt was not affected (Fig 3C). Dephosphorylation of 4E-BP1 was analyzed by detecting nonphosphorylated Thr46 residues on 4E-BP1 in the rapamycin treated cells. Rapamycin did not inhibit 4E-BP1 hyperphosphorylation at any of the four residues in NDV-infected cells (Fig 3D). By contrast, phosphorylation of p70S6K and rpS6 was inhibited substantially under the same conditions, suggesting the involvement of an alternative regulatory mechanism of 4E-BP1 phosphorylation by NDV infection. These results suggest a differential regulation of 4E-BP1 mediated by NDV, and indicate that NDV provides resistance to rapamycin-mediated inhibition of host translation initiation.

NDV infection activates an unknown mechanisms to maintain 4E-BP1 phosphorylation even if mTOR kinase is inhibited, it was hypothezised that treatment with rapamycin could not inhibit the NDV induced eIF4F complex assembly. In order to prove that, HeLa cells were mock-treated or infected with NDV for 8 h in the presence or absence of rapamycin. eIF4F complexes prepared from the lysates were analyzed by m^7^GTP pull-down and Western blot analysis as performed previously. Although 4E-BP1 loading to eIF4F complexes was slightly enhanced in rapamycin-treated cells, rapamycin did not significantly alter the amount of eIF4G bound to eIF4E (Fig 3E and 3F). We then determined the effect of rapamycin on viral protein synthesis in NDV infected HeLa cells. As expected, rapamycin treatment did not affect the synthesis of viral proteins or production of infectious virus (Fig 3E and 3G). These data suggest that NDV infection activates an unknown mechanism to maintain the integrity of the eIF4F complex even if mTOR kinase is inhibited.

### Activation of p38 MAPK/Mnk1 pathway induced eIF4E phosphorylation and inhibited 4E-BP1 phosphorylation in response to rapamycin treatment

**Fig 4:**
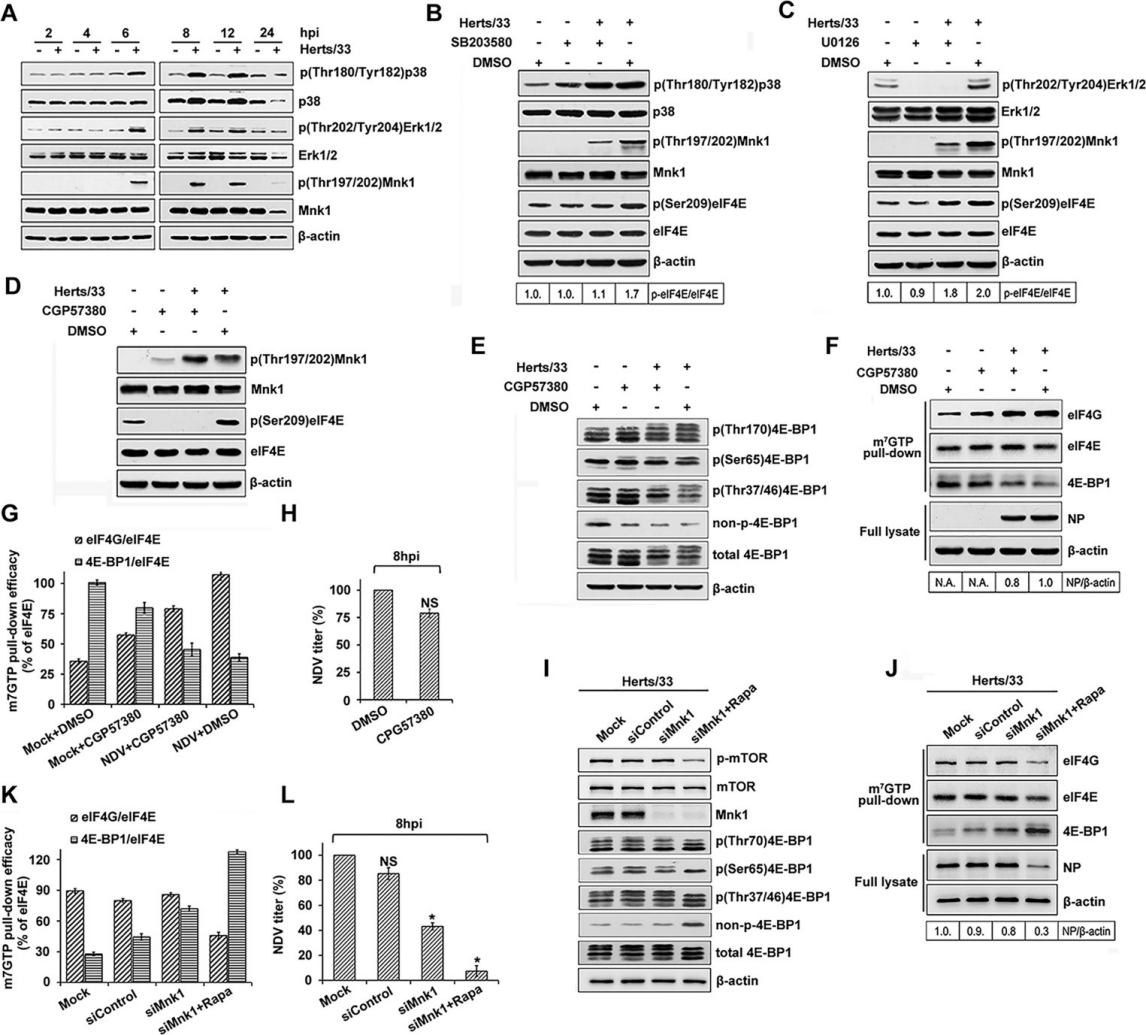
Activation of p38 MAPK/ Mnk-1 pathway induces eIF4E phosphorylation, but is not essential for viral protein synthesis. (A) Phosphorylation of p38, Erk1/2 and Mnk-1 is stimulated in NDV infected HeLa cells. HeLa cells were either mock-infected or infected with NDV (5MOI). At the indicated times (hpi), total protein was isolated, and equivalent amounts were fractionated by SDS-PAGE, and analyzed by immunoblotting using antibodies as shown. (B-D) eIF4E phosphorylation results from the activation of p38, but not Erk, in NDV-infected HeLa cells. Samples (as described in A) isolated from cultures treated with either DMSO, the p38 inhibitor SB203580 (B), the MEK1/2 inhibitor U0126 (C) or CGP 57380 (D) were analyzed by Western blotting as in (A). The band intensities of p-eIF4E was determined and normalized to eIF4E. The intensity bind ratio for uninfected cells in the absence of SB203580 (B) and U0126 (C) was given a value of 1 and indicated in the frames below the blots. (E) Phosphorylation of 4E-BP1 induced by NDV is not mediated by p38/Mnk-1 pathway. HeLa cells were pretreated 2 h with 10 μM SB203580 or CGP57380 and infected or mock-infected in the presence of inhibitors for 8 h before cells were harvested. The phosphorylation status of 4E-BP1 was analyzed using antibodies against total 4E-BP1, phopho-4E-BP1 (Thr70), phopho-4E-BP1 (Ser65), phopho-4E-BP1 (Thr37/46) and non-phospho-4E-BP1 (Thr46). (F) eIF4F complex loading is not affected by eIF4E dephosphorylation. eIF4F complexes were precipitated from protein lysates described in (A) by m7 GTP pull-down assay. eIF4G, eIF4E, 4E-BP1, NP and β-actin were detected in eluted or full lysate by Western blotting. The band intensities of NP was determined and normalized to β-actin. The intensity bind ratio for infected cells in the absence of CPG57380 was given a value of 1 and indicated in the frames below the blots. (G) The efficacy of cap Sepharose pull-down in the presence of CGP57380 was determined using immunoblotting for eIF4E, eIF4G and 4E-BP1. The efficacies of eIF4G and 4E-BP1 pull-down are expressed and normalized as the percentage of the eIF4E. (H) The extracellular virus yields were determined by TCID50 assay at 8 hpi and expressed as percentage of control. Data are presented as means from three independent experiments. Significance is analyzed by two-tailed Student's t-test (*, p<0.05; ns, not significant). (I) Mnk1 kinase was depleted in HeLa cells using siRNA. At 48 h post transfection, the cells were infected with NDV at 5 MOI in the presence of rapamycin or a DMSO solvent control and harvested at 8 hpi. Total protein was isolated, and equivalent amounts were fractionated by SDS-PAGE, and analyzed by immunoblotting using antibodies as shown. (J) Western blot analysis for eIF4F assembly of m^7^GTP precipitates HeLa cells that were infected with 5 MOI NDV in the presence of rapamycin or a DMSO solvent control after transfection with duplex siRNA oligonucleotides against MNK1 or control siRNA. NP and β-actin were detected in full lysate by Western blotting. The band intensities of NP was determined and normalized to β-actin. The intensity bind ratio for mock cells was given a value of 1 and indicated in the frames below the blots. (K) The efficacy of cap Sepharose pull-down was determined using immunoblotting for eIF4E, eIF4G and 4E-BP1. The efficacies of eIF4G and 4E-BP1 pull-down are expressed and normalized as the percentage of the eIF4E. (L) The extracellular virus yields were determined by TCID50 assay at 8 hpi and expressed as percentage of control. Data are presented as means from three independent experiments. Significance is analyzed by one-way analysis of variance followed by Dunnett’s test (*, p<0.05; ns, not significant).

Since mTOR activity did not affect eIF4E phosphorylation during NDV infection, we next investigated the role of Mnk1 signaling pathway on eIF4E phosphorylation in NDV infected cells. Western blot analysis showed significantly enhanced Mnk1 phosphorylation at Thr197/202 at 6, 8 and 12 hpi (Fig 4A), which correlated with a significant increase in eIF4E phosphorylation (Fig 1E). Mnk1 can be activated by either p38 or Erk kinases [46]. Subsequently, we examined the activation of p38 and Erk kinases in NDV-infected HeLa cells and observed increased phosphorylation of p38 and Erk at 6, 8 and 12 hpi (Fig 4A). This demonstrates that two kinases required for Mnk1 signaling were activated in NDV-infected cells. Furthermore, we investigated whether p38 and Erk acted as an upstream activator for NDV-induced phosphorylation of eIF4E. Treatment of HeLa cells with a p38 inhibtor SB203580 and Erk upstream target MEK1/2 inhibitor U0126 led to significant suppression of NDV-mediated Mnk1 phosphorylation (Fig 4B and 4C). The addition of SB203580 prevented phosphorylation of eIF4E in NDV infected HeLa cells, whereas U0126 did not (Fig 4B and 4C). It should be noted that SB203580 inhibits p38 catalytic activity by competing with ATP for its binding site, but does not inhibit phosphorylation of p38 (Fig 4B) [47]. The Mnk1 inhibitor CGP57380 also effectively prevented eIF4E phosphorylation (Fig 4D). However, it did not induce the obvious dephosphorylation of 4E-BP1 (Fig 4E). Thus, these results demonstrated that although both upstream kinases ERK and p38 are activated, only the activation of p38 is required for NDV-induced phosphorylation of eIF4E.

To further investigate the role of Mnk1 on eIF4F assembly and NDV replication, we used CGP57380 to inhibit Mnk1 function. No influence was shown on the assembly of eIF4F complexes (Fig 4F and 4G), suggesting that eIF4E is phosphorylated by Mnk1 once assembled into the eIF4F complex [16, 44]. We proceeded to evaluate the importance of eIF4E phosphorylation on NDV replication in the presence and absence of CGP57380. No detectable changes of viral NP protein were observed after inhibitor treatment (Fig 4F). Moreover, preventing eIF4E phosphorylation with CGP57380 only resulted in a 25% decrease in viral yields and was not deemed significant (Fig 4H).

More recently, 4E-BP1 phosphorylation and the association of 4E-BP1 with eIF4E were found to be regulated via the activated Mnk1 signaling pathway [48]. We found that CGP57380 blocks Mnk1 and eIF4E phosphorylation in NDV-infected cells. To further characterize the effect of Mnk1 kinase on the NDV-induced rapamycin-insensitive phosphorylation of 4E-BP1, Mnk1 kinase was depleted in HeLa cells using siRNA. Western blot analysis showed no significant changes in NDV induced 4E-BP1 hyperphosphorylation after Mnk1 depletion, whereas combined rapamycin treatment resulted in a obvious inhibition of 4E-BP1 phosphorylation (Fig 4I). Targeting Mnk1 with siRNA in combination with rapamycin treatment also markedly inhibited the eIF4F comlpex assembly level in m^7^GTP-precipitated complexes from NDV-infected HeLa cells (Fig 4J). Accordingly, viral protein synthesis and virus yields in Mnk1-depleted and rapamycin treated cells at 8 hpi were significantly lower than those in cells with Mnk1 siRNA transfection or rapamycin treatment respectively (Fig 4K and 4L). Based on these observations, we concluded that NDV infection induced activation of the Mnk1 signaling pathway functioned not only eIF4E phosphorylation but also maintainance of 4E-BP1 phosphorylation and viral protein synthesis in response to rapamycin treatment.

### NDV NP protein interacted with eIF4E to inhibit host for viral mRNA translation

**Fig 5:**
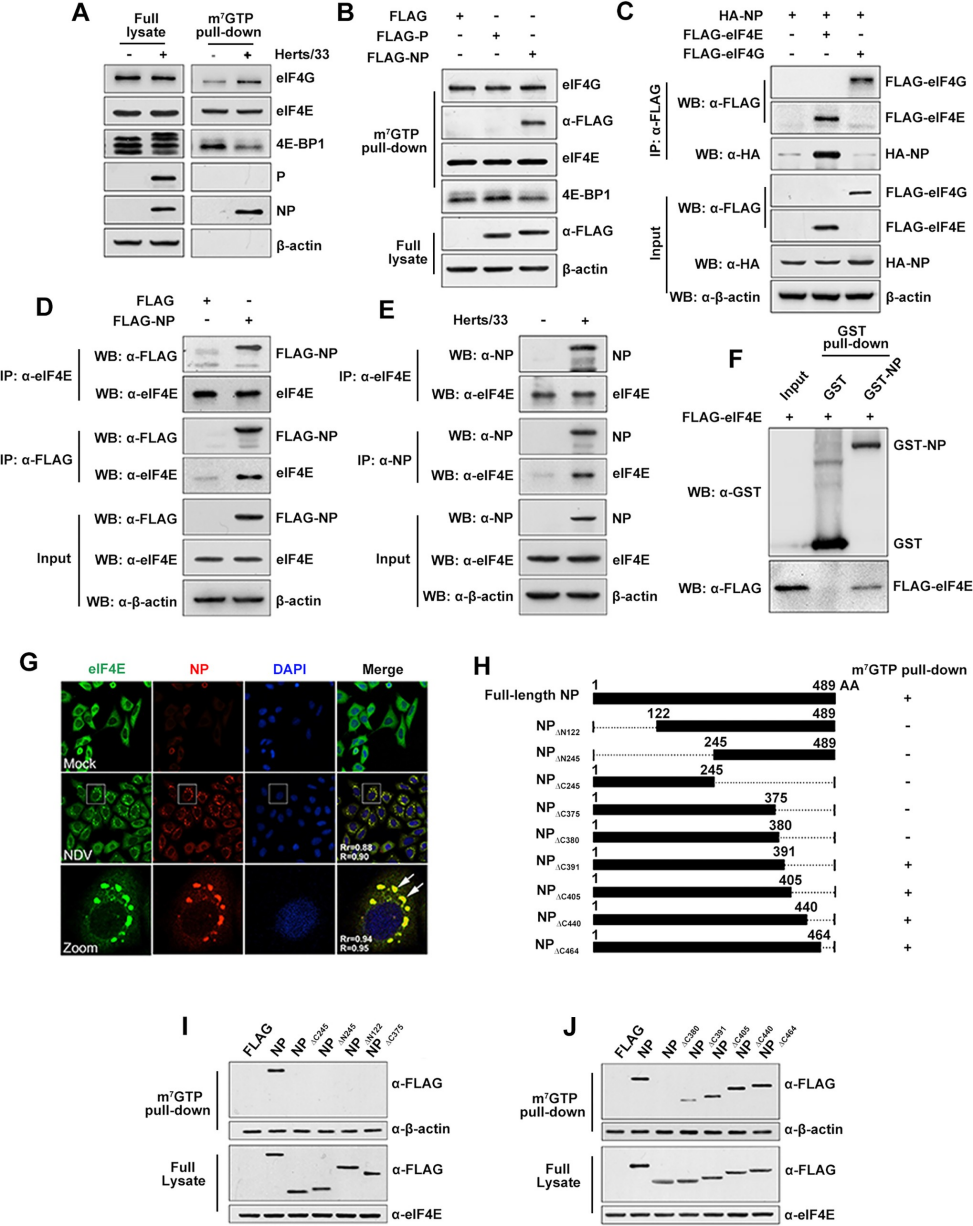
NDV NP protein physically interacts with eIF4E. (A) m^7^GTP pull-down of NP with eIF4F complex. HeLa cells infected with 5MOI of NDV Herts/33 were harvested at 8 hpi, lysates prepared and eIF4F complex were precipitated by m^7^GTP pull down assay. The precipitates were boiled in 2×loading buffer and the component proteins were analyzed by western blotting for the detection of P, NP, eIF4E, eIF4G 4E-BP1 and β-actin. (B) Association of NP with eIF4F in vitro. HeLa cells were transfected with the indicated plasmids or empty vectors, and the whole cell lysates obtained at 48 hpi. Proteins precipitated with m7GTP sepharose beads were detected by immunobloting with specific antibodies against FLAG, eIF4E, eIF4G 4E-BP1 and β-actin. (C) 293T cells were transfected with 3×FLAG tagged eIF4E or eIF4G and HA-tagged NP. These cells were harvested after transfection for 48 h. The interaction between NP and eIF4E was confrmed by co-IP with an anti-FLAG antibody and immunoblotting with an anti-HA antibody. (D) Reciprocal co-precipitation of NP with endogenous eIF4E. Cell lysates expressing FLAG-NP were immunoprecipitated with an FLAG or eIF4E antibodies and immunobloted with specific antibodies as mentioned. (E) Co-IP of NP protein with endogenous eIF4E during NDV infection. NDV-infected (+) or mock-infected (-) HeLa cells were used for IP with anti-NP protein or eIF4E antibody and immunoblotted with the indicated antibodies. (F) GST pulldown assay. Glutathione beads conjugated to GST or the GST-NP fusion protein were incubated with eIF4E overexpressing cell lysate. After washing, proteins were eluted from the beads and SDS-PAGE was performed. The presence of eIF4E was detected by immunoblotting with anti-Flag antibody. GST and GST-NP protein expression was confirmed by immunoblotting with anti-GST antibody. (G) eIF4E was redistributed and colocalized with NDV NP protein.HeLa cells were seeded on glass coverslips and mock-infected or infected with NDV Herts/33 at an MOI of 5. At 8 hpi cells were fixed and stained with anti-eIF4E and NP antibodies and then visualized by confocal microscopy. (H) Schematic representation of the deletion mutants of NP protein. Square frames represent the protein product of each truncated NP gene and the amino acid positions are indicated upon the frames. Dotted lines indicate deleted regions. (I, J) The N-terminal 391 residues of NP are sufficient to allow a heterologous protein to associate with eIF4E. Multiple C-terminal and N-terminal truncations of Flag-tagged NP were expressed in HeLa cells respectively. eIF4E were isolated by adsorption to 7-methyl GTP Sepharose beads, fractionated by SDS-PAGE, and analyzed by immunoblotting with the specific antibody.

Viral proteins have been shown to bind to eIF4F complex proteins to function on translation [45, 49], we further investigated whether NDV viral proteins play similar roles. We purified the eIF4F complex and associated viral protein from NDV-infected and mock-treated cells using 7-methyl GTP Sepharose beads, which binds to eIF4E and therefore can be used to enrich eIF4F-interacting proteins. NP was detected from the eIF4F complex of NDV-infected cells, but not from that of the mock-treated cells (Fig 5A), suggesting its physical interaction with eIF4F complex proteins. Both eIF4E and eIF4G were detected in the complex, but the NDV P protein and β-actin were not detected by m^7^GTP pull-down assay, confirming the specific interaction of NP with eIF4F in infected cells (Fig 5A). To further characterize the association of viral NP protein with eIF4F, we performed m^7^GTP pull-down assays with HeLa cells expressing NDV NP and P proteins, and we found that eIF4F complexes selectively interacted with NP but not P protein (Fig 5B). These data demonstrate that NP can specifically bind to eIF4F complexs.

To identify the specific component of eIF4F complex that potentially associate with the NDV NP protein, co-immunoprecipitation (Co-IP) experiments were performed with 293T cells transiently coexpressing HA-tagged NP and FLAG tagged eIF4E or eIF4G protein. Co-IP with showed that NP formed a complex with eIF4E but not eIF4G or FLAG tag (Fig 5C). To determine whether NP protein interacts with eIF4E in NDV-infected cells, the NDV-infected cell lysates were immunoprecipitated with NP protein antibodies. Both NP and eIF4E proteins were detected in NDV-infected cells (Fig 5D and 5E), indicating that NP interected with eIF4E during NDV infection. Moreover, a GST pull-down assay was performed with glutathione beads conjugated to GST-NP or GST tag protein to validate NP-eIF4E interactions. Flag-eIF4E from cell lysates was pulled-down by GST-NP but not GST bound beads, suggesting that NP directly binds to eIF4E (Fig 5F). Furthermore, the co-localization of eIF4E and NP protein in NDV-infected cells was examined using confocal fluorescence microscopy. eIF4E was diffusely dispersed throughout the cytoplasm of mock-treated HeLa cells, however, at 8 hpi, it was clustered and co-localized extensively with NDV-NP (Fig 5G), suggesting that NDV infection induced eIF4E redistribution and co-localization with NP protein. To determine which NP domain is necessary for the interaction with eIF4E, several NDV NP truncation mutants were generated (Fig 5H) and expressed in HeLa cells. Cells transfected with different mutants were harvested and evaluated using m^7^GTP pull-downs. The results showed that both the NP N-terminal deletion mutants (NP_ΔN122_ and NP_ΔN245_) and C-terminal deletion mutants (NP_ΔC245_ and NP_ΔC375_) lost its ability to interact with eIF4F complex (Fig 5I). Furthermore, NP_ΔC464_, NP_ΔC440_, NP_ΔC405_ and NP_ΔC391_, but not NP_ΔC380_, asssociated with the eIF4F complex (Fig 5J), collectively suggesting 1–380 amino acids (aa) were necessary for interaction between NP and eIF4F complex. Interestingly, the interaction ability between eIF4F complex and two deletion mutants (NP_ΔC391_ and NP_ΔC405_) was decreased compared with other mutants, indicating this domain (aa 380–405) was also important, although not essential, for NP-eIF4F interaction.

### NDV NP protein was involved in the inhibition of host translation and selective translation of viral mRNA

**Fig 6:**
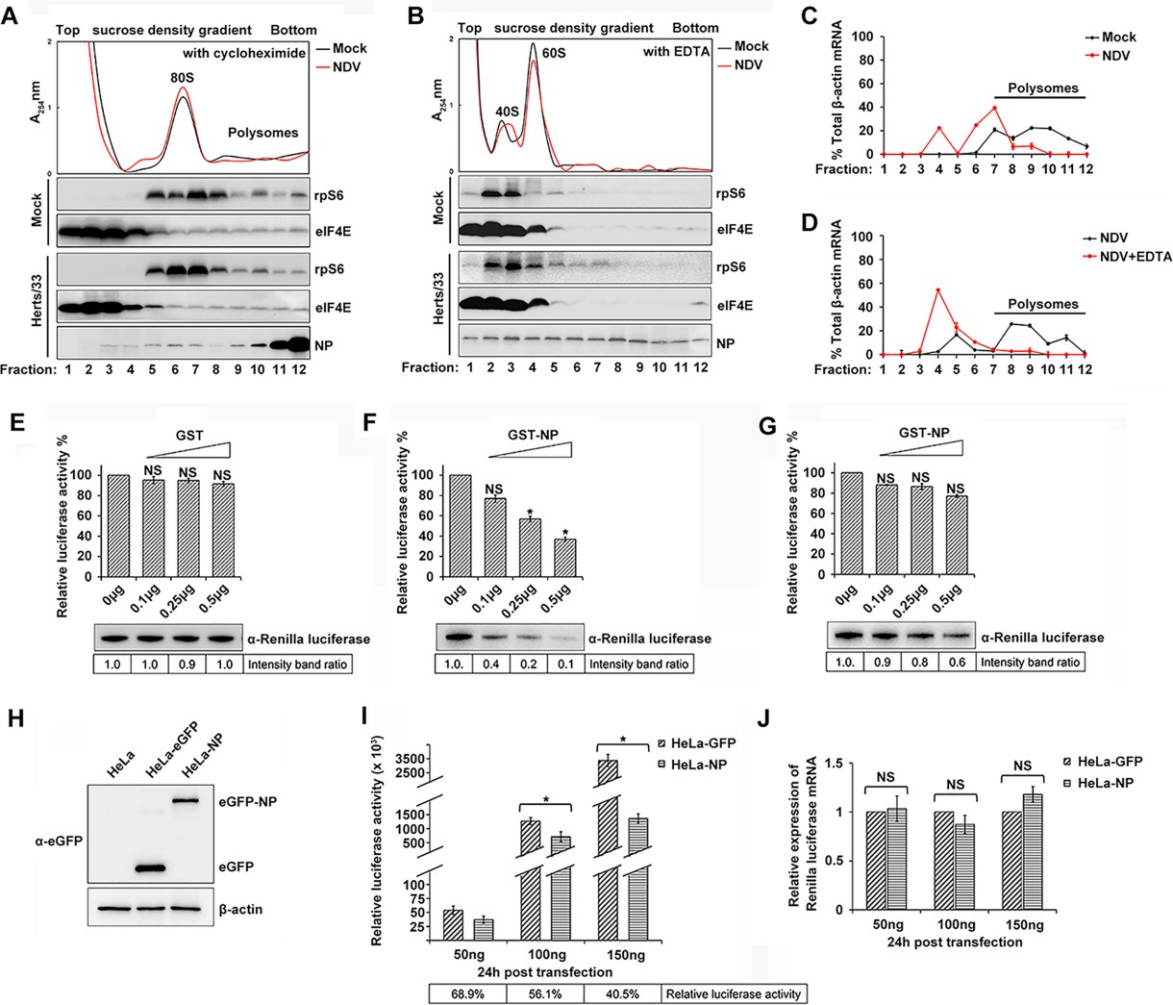
NDV NP protein fractionates with polysomes and inhibits protein synthesis in vitro and in vivo. (A) Upper panel: total cytoplasmic ribosomes (from lysates of NDV-infected and mock-infected HeLa cells) were fractionated by sucrose density gradient centrifugation. The ribosome profiles were obtained by measuring the absorbance at 254 nm of individual fractions. Cycloheximide was present in each sample. Lower panels: fractions were immunoblotted using the antibodies indicated on the right. (B) Ribosome profiles of NDV-infected and mock-infected HeLa cell extracts treated with 20mM EDTA to dissociate polysomes. Fractions were immunoblotted using the antibodies indicated on the right. (C) Distribution of β-actin mRNA with ribosomal complexes in NDV-infected HeLa cells. Transcript number, as determined by quantitative RT-PCR, is expressed as the percentage of total β-actin transcripts recovered and plotted against fraction number. The results are given as the mean ± SD from a representative quantitative RT-PCR experiment performed in duplicate. (D) Distribution of NDV NP mRNA with ribosomal complexes in NDV-infected HeLa cells treated with 20mM EDTA. Lysates were resolved and fractionated, and mRNA distribution was determined as in C. (E, F) Increasing amounts of recombinant GST (E) and GST-NP (F) were incubated with rabbit reticulocyte lysates. A capped reporter, RNA encoding the Renilla luciferase was added and translated. Translation products were detected by measuring the activity of Renilla luciferase and Western blot analysis. The translation efficiency of Renilla luciferase is expressed as a percentage of the control set to 100%. Results show the means±S.E. from three separate experiments. Significance was analyzed by one-way analysis of variance followed by Dunnett’s test (*, p<0.05; ns, not significant). The intensities of Renilla luciferase bands were quantified by densitometric analysis and indicated below the blots. (G) A reporter mRNA was preincubated with increasing amount of GST-NP and then added to rabbit reticulocyte lysates. Translated products were quantified in a similar manner as panel E and F. (H) NDV-NP protein was expressed in HeLa cells by lentiviral packaging system and the expression of NP was detected by Western blotting. (I, J) A reporter plasmid encoding the Renilla luciferase gene was transfected with increasing concentrations in HeLa-GFP and HeLa-NP cells respectively. Cells were harvested, an aliquot was lysed, and Renilla luciferase activity was measured by using a luminometer (I). The relative luciferase activity was indicated in the frames below the graphics. Another aliquot was harvested, and the total RNA was analyzed for Renilla luciferase transcripts using real-time RT-PCR. The Renilla luciferase mRNA levels in HeLa-NP cells are expressed relative to those in HeLa-GFP cells (J). The results shown represent means of three experiments. Significance is analyzed by two-tailed Student's t-test (*, p<0.05; ns, not significant).

Demonstrating the recruitment of eIF4E-associated NDV-NP to polysomes during NDV infection would confirm its involvement in host protein translation. For this purpose, cell extracts from NDV-infected and mock-treated HeLa cells were subjected to a 7–47% sucrose density gradient ultracentrifugation, and the fractions were analyzed by absorbance determination at 254 nm. We found that NDV-infected cells exhibited a moderate translational defect as shown by a slight decrease in the amount of polysomes (Fig 6A), indicated that a small portion of free ribosomal subunits no longer participates in mRNA translation during NDV infection, which could suggest a moderate inhibition of host global protein synthesis. To assess the distribution of NP protein in the gradient fractions, proteins from each fraction were acetone-precipitated and analyzed by Western blot. NP protein was detectable throughout the gradient and reached peak at fraction 12 in polysomal fractions of NDV-infected HeLa cells (Fig 6A), confirming its involvement in host cap-dependent translation. eIF4E and ribosomal S6 proteins were also present in both the monosomal and the polysomal fractions confirming the integrity of the fractions. In order to verify the association of NDV-NP with polysomes, cell extracts were treated with 20 mM EDTA prior to being loaded on the gradient, resulting the dissociation of polysomes into its component free 40S and 60S subunits (Fig 6B). EDTA treatment effectively abolished the association of NDV-NP with polysomes and induced NDV-NP to shift into lighter fractions, suggesting that NDV-NP does not sediment with high density complexes in the absence of polysomes. These results suggest that NDV-NP is actively involved in host cap-dependent translation and contributes to the viral life cycle by relocating to polysomes during NDV infection.

Moreover, we investigated whether the NP functions on host and virus mRNA expression during NDV infection. HeLa cells were NDV-infected or mock-treated, and cell lysates were resolved on a 7–47% linear sucrose gradient. Each fraction was ethanol-precipitated for quantitative RT-PCR analysis. Our results revealed that the transcripts of cellular β-actin were distributed throughout the separate monosomal and polysomal fractions of both NDV-infected and mock-treated cells, but there was a considerable shift to lighter fractions following NDV infection (Fig 6C). In mock-treated HeLa cells, 80% of total β-actin mRNAs was in polysome-bound fractions (Fig 6C, Mock, fractions 8 to 12), and only some of the portions were present in fractions containing monosomes (Fig 6C, Mock, fractions 4 to 7). In contrast, NDV infection resulted in an arrest of β-actin mRNAs in the monosome peaks. Different from host β-actin mRNA, the distribution of NDV NP mRNA gradually increased from monosome to polysome, suggesting an increased efficiency of ribosome loading and continued translation (Fig 6D, NDV, fractions 4 to 12). For a control, polysomes were dissociated with 20 mM EDTA. This treatment caused NDV NP mRNA to shift from the bottom to the top of the gradient (Fig 6D, NDV+EDTA, fractions 3 to 6), confirming the association of polyribosomes with viral mRNAs. Similar results were consistently achieved in three separate experiments. This analysis clearly showed that NDV infection inhibited host mRNAs but enhanced viral mRNA loading in polyribosomes.

To examine whether the NDV NP protein functions on the shutoff of host cellular protein translation, we investigated the effect of NP protein on eIF2α phosphorylation. As shown in S5 Fig, NP overexpression, as well as NDV infection and thapsigargin treatment (a known ER stress inducer) led to the significant phosphorylation of eIF2α. The effect of NDV NP protein on host protein synthesis was monitored by an *in vitro* system. GST-NP markedly inhibited capped reporter mRNA translation in a dose-dependent manner, whereas GST tag protein did not interfere with the translation reaction (Fig 6E and 6F). In addition, preincubation of capped reporter mRNA with GST-NP at 30°C for 90 min before adding to the rabbit reticulocyte lysate system only had a little effect on Renilla luciferase translation (Fig 6G), indicating that the suppression of translation by GST-NP did not result from nonspecific binding between the capped reporter mRNA and GST-NP. The NDV NP protein functions on the host translation suppression was further examined on the NP expressing HeLa cells by lentiviral packaging system. HeLa cells stably expressing eGFP-NP and eGFP (negative control) were confirmed by Western blot (Fig 6H). As expected, the Renilla luciferase synthesis was suppressed in HeLa-NP cells, comparing with that in HeLa-eGFP cells (Fig 6I). There was no difference in the rate of Renilla luciferase mRNA transcription between HeLa-NP cells and HeLa-eGFP cells as measured by RT-qPCR (Fig 6J). Therefore, the suppression of Renilla luciferase activity by NDV-NP is suggested to be caused by inhibition of the translation.

## Discussion

During NDV infection, host cellular protein synthesis is almost completely inhibited accompanied by PKR-induced phosphorylation of eIF-2α, while viral mRNAs are translated efficiently [50, 51]. Contrary to cellular mRNA, NDV mRNA was more resistant to the effect of phosphorylated eIF2a and was translated preferentially even when phosphorylated eIF2a was dramatically accumulated. This prompted us to investigate the mechanisms for the selective synthesis of viral protein in the shutoff stage during NDV infection. We focused on the canonical responses to virus infection, which regulate the translation machinery through concomitant regulation of multiple signaling pathways together with the assembly of eIF4F complexes and the hyperphosphorylation of the translational repressor 4E-BP1. We found that NDV adopted multiple strategies to activate the host translational machinery, to benefit the progeny viruse production and to inhibit the cellular protein synthesis.

The regulation of eIF4F assembly is a fundamental step in controlling cap-dependent translation initiation in eukaryotes. Normally, hyperphosphorylation of the 4E-BP1 translational repressor by mTOR releases the cap-binding protein eIF4E, allowing eIF4E to associate with eIF4G and promote eIF4F complex formation [52]. Active mTOR also promotes eIF4F assembly and cap-dependent translation initiation by phosphorylating eIF4G. Translational control via the mTOR pathway plays important roles in cell growth and proliferation. Several viruses manipulate mTOR pathways to alter 4E-BP1 phosphorylation and eIF4F complex availability to favor translation from their own mRNAs. For example, both African swine fever virus and Herpes simplex virus type 1 activate mTOR to promote 4E-BP1 phosphorylation and eIF4F assembly, whereas Hepatitis C virus NS5A binds to the eIF4F complex and up-regulates host translation initiation machinery through regulation of Akt/mTOR pathways [35, 46, 53]. Here, our data show that NDV induces a PI3K-dependent mechanism that drives the phosphorylation of 4E-BP1 in order to ensure the integrity of eIF4F complex and enhance viral protein production even when mTOR is inhibited. The m^**7**^GTP pull-down experiments support this hypothesis, showing that the mTOR-independent phosphorylation of 4E-BP1 correlates with inhibition of eIF4E binding to 4E-BP1 and the promotion of its association with the eIF4F complex. In agreement, a wide range of viruses were also reported to stimulate 4E-BP1 phosphorylation and enhance viral protein synthesis via a mTOR-independent mechanism. Both human cytomegalovirus and Hepatitis C Virus infection can induce mTOR-independent phosphorylation of 4E-BP1 in PI3K-dependent manner [35, 54]. Controlling the rapamycin-insensitive phosphorylation of 4E-BP1 could therefore be a more general mechanism that viruses use to maintain the integrity of the eIF4F complex to ensure cap-dependent translation regardless of the cellular conditions.

In addition to inactivating the repressor 4E-BP1, NDV infection activates p38 MAPK and its downstream target, the eIF4G-associated eIF4E-kinase Mnk1. eIF4E activity is regulated at multiple levels, including expression levels, modification of its regulatory proteins and the phosphorylation status of eIF4E. The MAPK-interacting kinases Mnk1 and its essential upstream activators Erk and p38 MAPK are shown to be involved in the phosphorylation of eIF4E on the conserved physiological site at serine 209 [55, 56]. Viruses have targeted the processes of eIF4E phosphorylation to facilitate translational selectivity for viral mRNAs during infection. Dephosphorylation of eIF4E occurs during infection with adenovirus, encephalomyocarditis virus, poliovirus and vesicular stomatitis virus, causing the shutoff of cellular protein synthesis [57–59]. In contrast, infection with herpes simplex virus 1, human cytomegalovirus and vaccina virus triggers Mnk1-dependent phosphorylation of eIF4E to enhance viral replication [46, 60, 61]. Although previous reports have shown that p38 MAPK and Erk are activated in NDV-infected cells to induce apoptosis [25, 62], the potential role of eIF4E phosphorylation in NDV infection has not been established. Here we showed that NDV infection induced the phosphorylation of Mnk1, correlated with the virus-induced hyperphosphorylation of eIF4E. Both Erk and p38 are activated in NDV-infected cells, however, the inhibitor experiments showed that only p38 activation is directly and required for eIF4E activation. Increased phosphorylated eIF4E abundance mediated by Mnk1 activity likely reflects eIF4F complex assembly on the newly minted or derepressed mRNAs terminus, as eIF4E phosphorylation occurs following eIF4F assembly when eIF4E is recruited to the eIF4G-Mnk1 complex [26]. While Mnk1 and eIF4E phosphorylation are proved not to be essential for NDV viral protein synthesis. These results are in agreement with previous reports that question the significance of eIF4E phosphorylation in its cap binding affinity [17, 63]. It is speculated that eIF4E phosphorylation may govern the translation to initiate on newly transcribed NDV viral mRNA rather than play any direct manipulation on NDV infection.

It is reasonable to speculate that other components of the eIF4F complex may also be modified by NDV infection to further ensure the integrity of the eIF4F complex even when mTOR is inhibited. A balance between the activity of the Akt/mTOR and the MAPK/Mnk1 pathway was reported in Prostate cancer cells, where suppression of one pathway was correlated with the activation of another, resulting in a defined translational level of specific mRNAs that supported cancer cell proliferation [64]. Combined pharmacological targeting of Mnk1 and mTOR shows greater growth inhibitory effects than the suppression of one of the pathways in many types of cancer cells [64]. In the latter studies, Mnk1 kinase was identified as a key regulator to sustain a level of protein synthesis via 4E-BP1 phosphorylation regulation in the absence of mTOR signaling in rapalog-treated glioma cells [48]. In the current study, we found that viral protein synthesis and eIF4F comlpex formation were markedly suppressed, accompanied by the efficient restraint of 4E-BP1 phosphorylation when both mTOR and Mnk1 were inhibited. Based on these observations, we concluded that during NDV infection, the activated Mnk1 pathway not only phosphorylates eIF4E but also contributes to the rapamycin-insensitive phosphorylation of 4E-BP1 followed by eIF4E dissociation to maintain continued eIF4F assembly and viral protein synthesis in response to mTOR inhibition. This suggests that a lack of mTOR signaling activates Mnk1-dependent compensatory mechanisms that maintain a level of protein synthesis. Should this hypothesis prove true, contributions by Mnk1 phosphorylation to translation control strategies is likely not to be confined to viral biology, but may also be important in cancer research.

A large number of viral infections exert a repressive effect on eIF4F complex, while a few studies have revealed a facilitatory role of viral proteins on eIF4F loading and viral mRNA selective translation [46, 65, 66]. The NP protein of paramyxoviruses plays a crucial role during replication of the genomic RNA, however, a limited number of studies have been carried out about the role of NP protein in viral mRNA translation. The measles virus (MeV) NP protein has the ability to bind to p40 subunit of eukaryotic translation initiation factor 3 (eIF3-p40; eIF3γ; eIF3H) and suppress mRNA translation [67]. In this study, eIF4E was identified as a NDV NP protein-binding protein by m7-GTP pull-down, coimmunoprecipitation and glutathione S-transferase pull-down analysis. eIF4E was found to be redistributed in NDV-infected cells to co-localize with viral protein at 8 hpi. These findings point to the idea that NDV activates and recruits eIF4E to areas where active viral translation takes place, and meanwhile decreases the availability of eIF4E for cellular mRNA translation and may contribute to host translation shutoff. Recent reports have revealed that a viral protein and several host factors regulate host protein synthesis by binding and redistributing eIF4E. For example, eIF4E is redistributed in cells infected with Reoviruses and vaccinia virus which replicate in the cytoplasm within discrete compartments termed viral factories (VF) [68, 69]. It could concentrate the host protein synthesis factors needed for translation of viral mRNA close to the regions of viral transcription, potentially favouring eIF4F assembly and sequestering translation factors from host mRNA to suppress host translation. Furthermore, Porcine sapovirus VPg protein is reported to be directly associated with eIF4E, facilitating viral protein synthesis[70]. Therefore, it is speculated that binding of NP to eIF4E facilitates, rather than restricts translation initiation and might result in enhanced selective translation of NDV viral mRNAs. In higher eukaryotes, mRNA is further methylated at the 2'-O position of the first ribose [71, 72]. NDV, in contrast, generates mRNAs lacking 2'-O-methylation [73]. mRNA cap structures play important roles in enhancing the translational efficiency as well as the stability of the mRNAs [74, 75], and 2'-O-methylation is known to increase the affinity of the cap structure for proteins such as eIF4E and other cap-binding proteins [71, 72]. In that case, NP protein might play a role in benefiting for the selective translation of viral mRNAs by binding to the eIF4E protein. Identification of specific domains on NP that interact with eIF4E, and the use of mutants or recombinant viruses that lack this interaction might in the future provide important information on the regulation of translation by NP in NDV infection.

Given the interaction of NDV-NP with eIF4E, NP protein was also proved to associated with polysomes during NDV infection. The association of viral protein with polysomes have been reported in several viruses. NS1 was proved to be co-localised with hStaufen protein in the polysomes of influenza virus-infected cells to stimulate translation by enhancing the rate of translation initiation [76, 77]. Similarly, it has been reported that the UL69 protein of Human cytomegalovirus, which is present on polysomes and associates with a variety of HCMV mRNAs, facilitates viral protein translation by associating with the mRNA cap-binding complex and excluding 4E-BP1 [78]. In our study, we observed an accumulation of NDV NP protein in polysome fractions of NDV-infected cells, and the association of NP with polysomes can be abolished by EDTA treatment. Thus, it appears that NDV-NP may actively involve in host cap-dependent translation by relocating to polysomes during NDV infection to control the selective translation of viral mRNAs. Supporting this hypothesis, we showed that the NDV NP mRNA is increased in the polysome fractions of NDV-infected cells, suggesting an increased efficiency of ribosome loading and continued translation of viral mRNA. In addition, host mRNAs were arrested in the monosome fractions, and formation of elongation-competent ribosomes on host mRNAs was completely suppressed. This analysis clearly shows that NDV infection inhibited the loading of polysomes to the host mRNAs and increase ribosome occupancy of viral mRNAs. Moreover, it is speculated that the recruitment of eIF4E associated NDV-NP polysome during NDV infection might result in enhanced selective translation of viral mRNAs.

The primary function of the NP/N protein of paramyxovirus is considered to form the viral helical nucleocapsid by encapsidating the viral genomic RNA, which serves as the template for viral transcription and replication. A previous study suggested that deletion of MeV N protein by specific siRNA dramatically represses the synthesis of viral mRNAs and genomic RNA[79]. Moreover, the association of MeV-N protein with eIF3-p40 is proved to be involved in the induction of host shutoff [67]. These findings indicate that the NP/N protein of paramyxovirus is important for virus replication. Our previous work has showed that transient expression of the NDV NP protein in HeLa cells induces ER stress via activation of UPR signaling through the PERK/eIF2α/ATF6 pathways, suggesting a prospective role of NP protein that involves the modulation of host translation mechanism [51]. In present study, we analyzed the potential inhibitory effect of NDV NP protein on host protein synthesis in cells. It is noteworthy that, suppression of transfected reporter gene in HeLa-NP cells was partial, the suppression rate reached nearly 40% inhibition. Our preliminary results indicate that the host *de novo* protein synthesis was almost completely abolished by NDV infection at 16 h postinfection, suggesting that additional mechanisms besides the association of NP with eIF4E are involved in NDV-mediated host shutoff. The molecular mechanism of selective viral mRNA translation under conditions of host protein shutoff is a multicomponent process that has been attributed to a variety of factors. Actually, we have confirmed that NDV infection activate PKR and trigger ER stress response, leading to eIF2α phosphorylation and translational shutoff [51]. Recently, we also found that NDV infection triggers stable formation of typical SGs through PKR/eIF2α pathway to restrict cellular mRNA translation, the preferential translation of viral mRNA was a result of translation factors and ribosome competition from an overwhelming abundance of viral mRNA [80]. Thus, the prominent host shutoff induced by NDV infection may be manipulated by multiple cooperative mechanisms. We hypothesize that the accumulation of phosphorylated eIF2a and the formation of typical SGs probably resulting from the activation of PERK or PKR occurs at the early stage of NDV infection and then binding of increased NP protein to eIF4E enhances the shutoff of host translation at later stage of infection. Moreover, based on the ability to suppress host mRNA translation in cells, NP relocating to polysomes could contribute to selective translation of viral mRNA or even specific cellular mRNA in NDV-infected cells. More studies such as profiling of the polysome-associated RNA population by microarray analyses would expand our understanding on these questions in detail.

**Fig 7:**
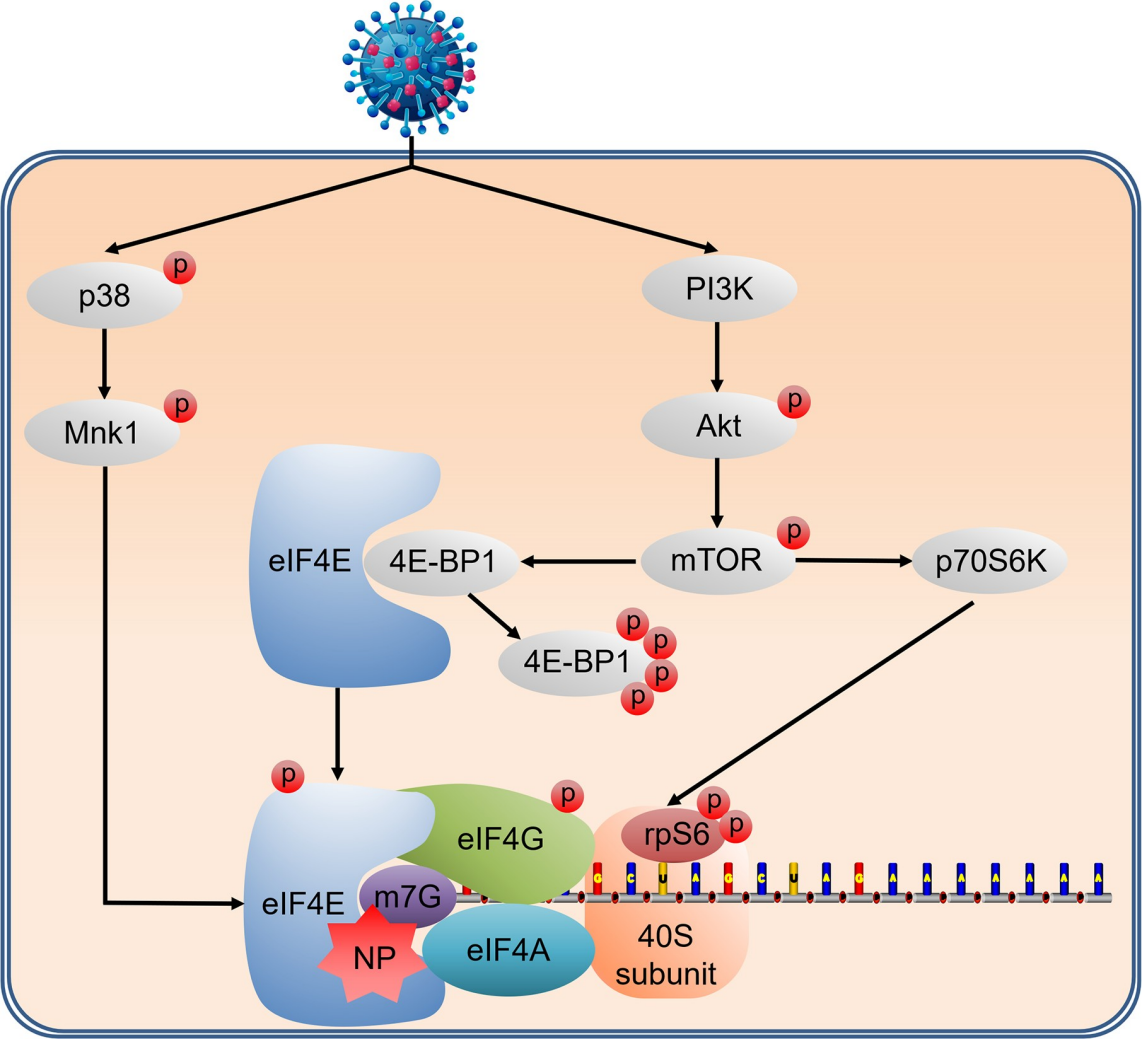
Proposed model for the cap-dependent translational control during NDV infection. NDV infection activates the host cap-dependent translation machinery to benefit viral translation and replication. NDV enhances eIF4E and eIF4G phosphorylation to promote eIF4F assembly and accelerate translation initiation by concomitant upregulation of PI3K/Akt/mTOR and p38 MAPK/Mnk1 pathways. Activated mTOR enhances translation initiation machinery through phosphorylation of the translational repressor 4E-BP1 and S6K1/rpS6. rpS6 phosphorylation may contribute to the sustained translation of host 5’TOP mRNAs which encode ribosomal protein, thereby facilitating the production of progeny viruses by supporting viral mRNA translation. In addition to phosphorylate eIF4E, p38 MAPK/Mnk1 signaling pathway also maintians 4EBP1 hyperphosphorylation and vrial protein synthesis to be resistant to rapamycin treatment. Finally, NDV NP protein can bind to eIF4E and facilitate the selective translation of viral mRNAs based on the involvement in cap-dependent translation during NDV infection by associating with polysomes and the ability to suppress host mRNA translation in vitro and in vivo.

In conclusion, our current study provides insight into how NDV manipulates host translation mechanisms to hijack host translation initiation machinery for facilitating viral replication (Fig 7). Our findings will help to provide new targets for antiviral drug discovery. Most importantly, understanding the mechanisms of translational control by NDV, an oncolytic virus, will help represent attractive new targets in the continuing quest for safe, effective cancer therapeutics.

## Supporting information

S1 Fig Cell viability under drug exposure by WST-1 assay. WST-1 assay showing cell viability after adding various concentrations of CGP57380 (A), Rapamycin (B), LY294002 (C), U0126 (D), SB203580 (E). Data are presented as means from three independent experiments. Significance is analyzed with two-tailed Student’s t test (*, P < 0.05; **, P < 0.01; #, P ˃ 0.05), compared to the vehicle group.
(TIF)

S2 Fig The inhibition of global protein synthesis in NDV-infected cells. (A) HeLa cells were infected with NDV Herts/33 at an MOI of 1. At various time points, the cells were pulse labeled with 1 μM puromycin for an hour before collection. Cell samples were then subjected to western blot analysis using anti-puromycin, anti-NP, or anti-β-actin antibody. (B) Representative results are shown with graphs representing the ratio of puromycin to β-actin normalized to the control condition (*, P < 0.05).
(TIF)

S3 Fig Inhibition of host protein synthesis in NDV-infected DF1 cells. (A) The growth curve of NDV in DF-1 cells. HeLa cells were infected with 5 MOI of Herts/33. Supernatants were harvested at indicated times and were subjected to TCID_50_ assay. TCID_50_ was calculated using Reed-Munch mathematical analysis. (B) DF-1 cells infected with Herts/33 were labeled with 100 mCi of [^35^S] methionine/cysteine for 1 h and collected at the indicated time. Labeled proteins were analyzed by SDS-PAGE followed by fluorography and autoradiography. Asterisks (*) indicate newly synthesized proteins detected only in Herts/33 infected cells. Molecular weight standards appear in the leftmost lane and their sizes (kDa) are indicated in the margin. Coomassie brilliant blue staining of the autoradiograph gel were performed to confirm the equivalence of protein loading. Quantitation of host protein synthesis in NDV-infected HeLa cells. The rates of protein synthesis were determined as fold changes of host protein synthesis in NDV-infected cells compared to that in mock-infected cells (lower panel). (C) DF1 cells were mock-infected or infected with NDV, and harvested at indicated times. Total protein was isolated, and equivalent amounts were fractionated by SDS-PAGE, and analyzed by immunoblotting using antibodies recognizing NP and β-actin.
(TIF)

S4 Fig UV-irradiation inactivated virus can not induce phosphorylation of eIF4G and eIF4E in HeLa cells. HeLa cells were either mock infected, or infected with UV-inactivated NDV at 5MOI at the indicated times following infection, total protein was isolated and fractionated by SDS-PAGE and analyzed by immunoblotting with the indicated antibodies.
(TIF)

S5 Fig The effect of NDV NP protein on eIF2α phosphorylation. HeLa cells were transfected with FLAG-NP plasmid, infected with NDV at an MOI of 5, or treated with 300 nM Tg for 24 h were harvested for Western blotting analysis of eIF2α, p-eIF2α, and β-actin. The intensities of phospho-eIF2α was determined by densitometry, normalized to total eIF2α.
(TIF)

S1 Table Primers used in this study. (DOCX)
