# Supplementary figures and images for: Newcastle Disease virus infection activates PI3K/Akt/mTOR and p38 MAPK/Mnk1 pathways to benefit viral mRNA translation via interaction of the viral NP protein and host eIF4E
Source: PLoS Pathog. 2020 Jun 30;16(6):e1008610. doi: 10.1371/journal.ppat.1008610 (PMC7326156; doi:10.1371/journal.ppat.1008610)

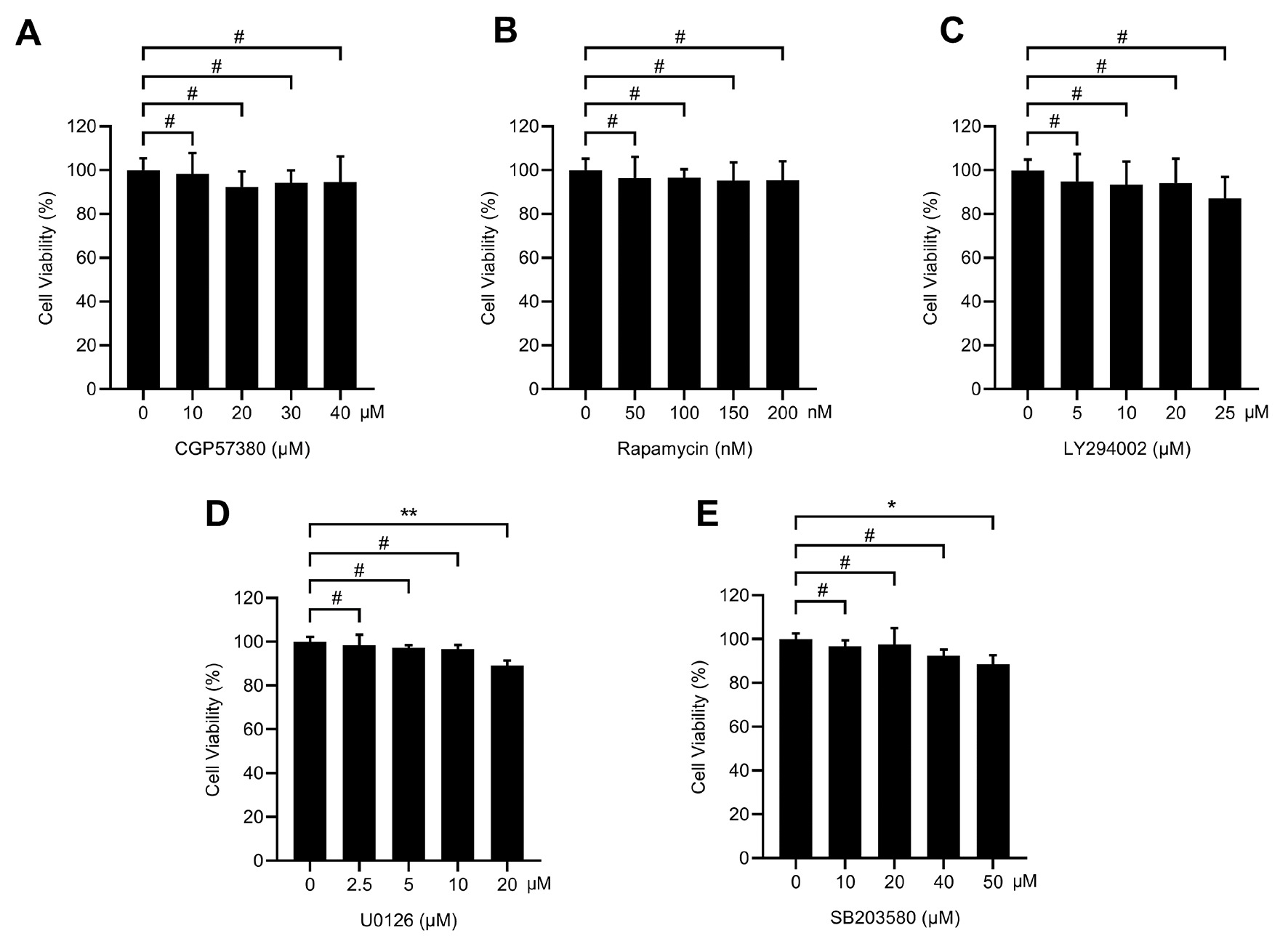

Supplement: S1 Fig — WST-1 assay showing cell viability after adding various concentrations of CGP57380 (A), Rapamycin (B), LY294002 (C), U0126 (D), SB203580 (E). Data are presented as means from three independent experiments. Significance is analyzed with two-tailed Student’s t test (*, P < 0.05; **, P < 0.01; #, P ˃ 0.05), compared to the vehicle group. (TIF) [file ppat.1008610.s001.tif]

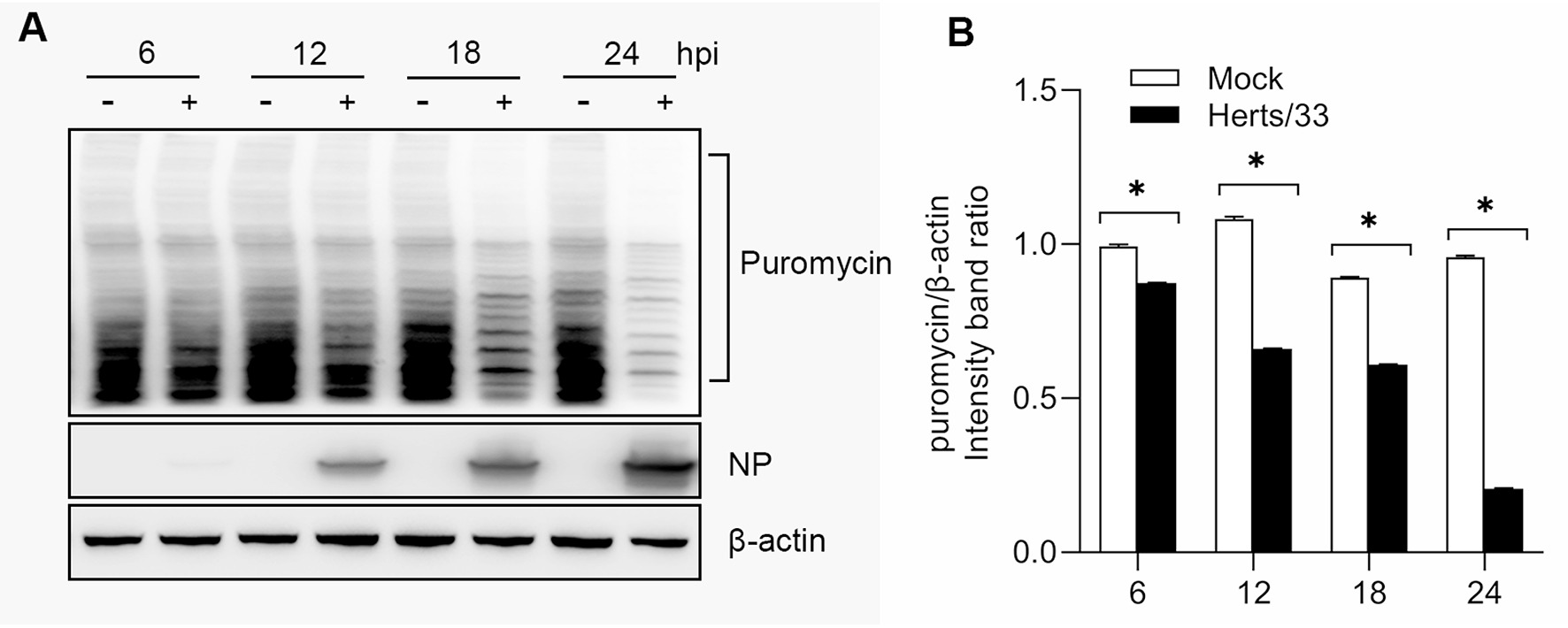

Supplement: S2 Fig — (A) HeLa cells were infected with NDV Herts/33 at an MOI of 1. At various time points, the cells were pulse labeled with 1 μM puromycin for an hour before collection. Cell samples were then subjected to western blot analysis using anti-puromycin, anti-NP, or anti-β-actin antibody. (B) Representative results are shown with graphs representing the ratio of puromycin to β-actin normalized to the control condition (*, P < 0.05). (TIF) [file ppat.1008610.s002.tif]

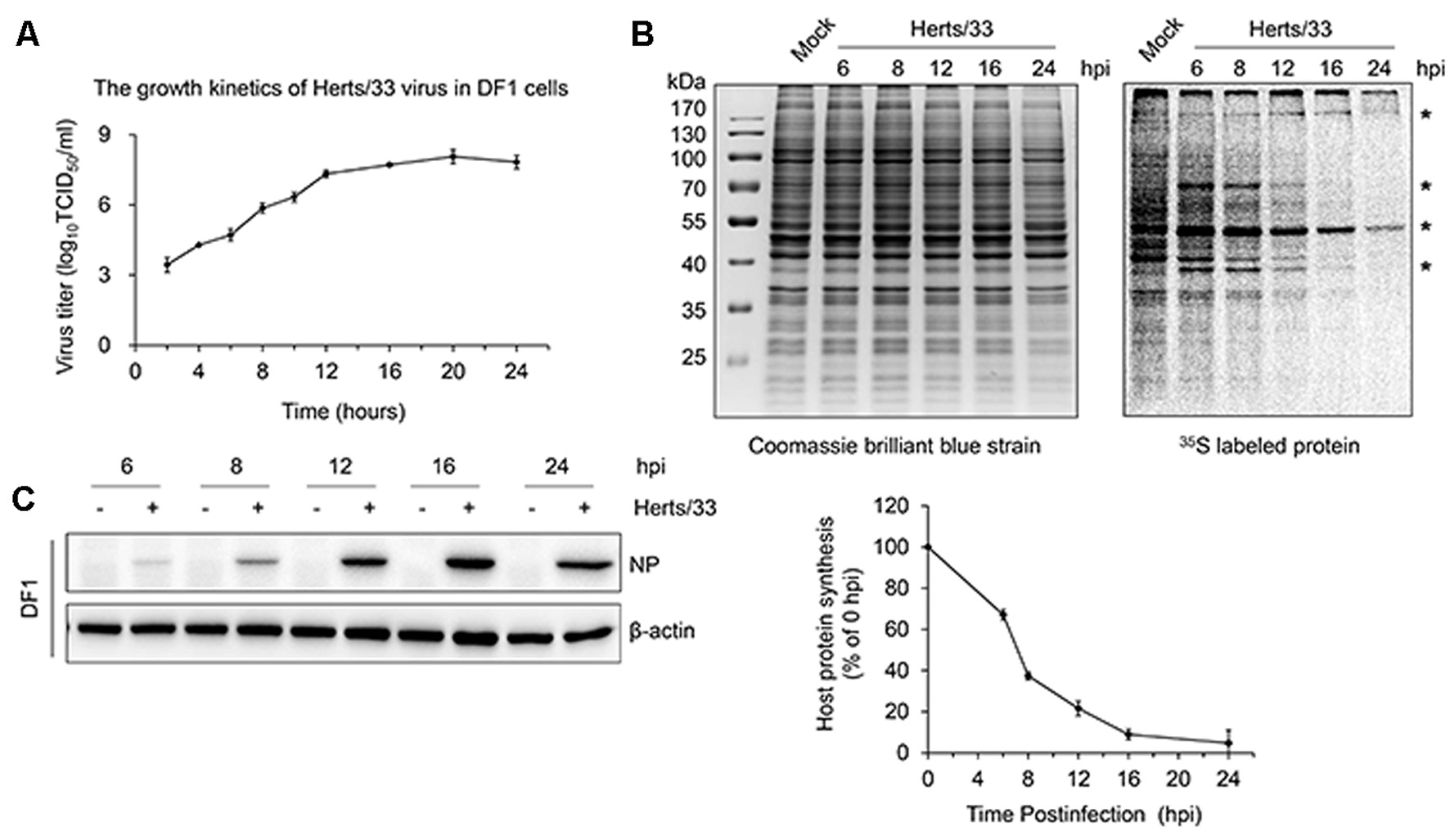

Supplement: S3 Fig — (A) The growth curve of NDV in DF-1 cells. HeLa cells were infected with 5 MOI of Herts/33. Supernatants were harvested at indicated times and were subjected to TCID50 assay. TCID50 was calculated using Reed-Munch mathematical analysis. (B) DF-1 cells infected with Herts/33 were labeled with 100 mCi of [35S] methionine/cysteine for 1 h and collected at the indicated time. Labeled proteins were analyzed by SDS-PAGE followed by fluorography and autoradiography. Asterisks (*) indicate newly synthesized proteins detected only in Herts/33 infected cells. Molecular weight standards appear in the leftmost lane and their sizes (kDa) are indicated in the margin. Coomassie brilliant blue staining of the autoradiograph gel were performed to confirm the equivalence of protein loading. Quantitation of host protein synthesis in NDV-infected HeLa cells. The rates of protein synthesis were determined as fold changes of host protein synthesis in NDV-infected cells compared to that in mock-infected cells (lower panel). (C) DF1 cells were mock-infected or infected with NDV, and harvested at indicated times. Total protein was isolated, and equivalent amounts were fractionated by SDS-PAGE, and analyzed by immunoblotting using antibodies recognizing NP and β-actin. (TIF) [file ppat.1008610.s003.tif]

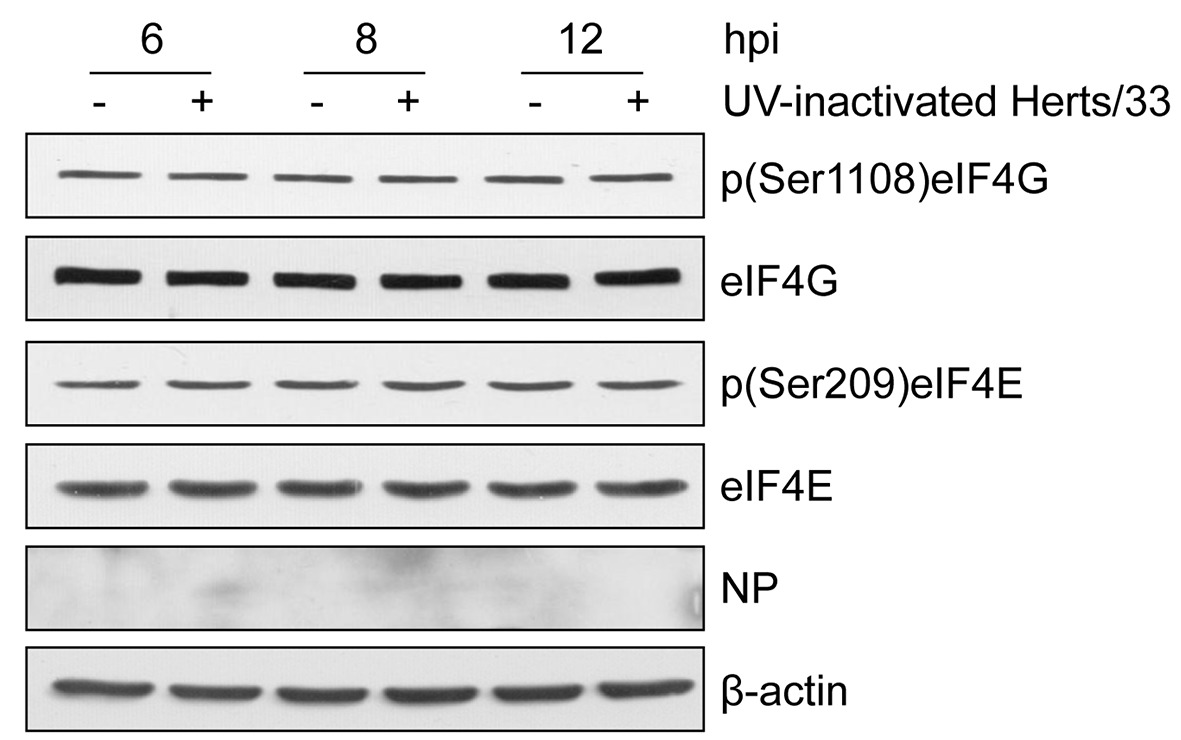

Supplement: S4 Fig — HeLa cells were either mock infected, or infected with UV-inactivated NDV at 5MOI at the indicated times following infection, total protein was isolated and fractionated by SDS-PAGE and analyzed by immunoblotting with the indicated antibodies. (TIF) [file ppat.1008610.s004.tif]

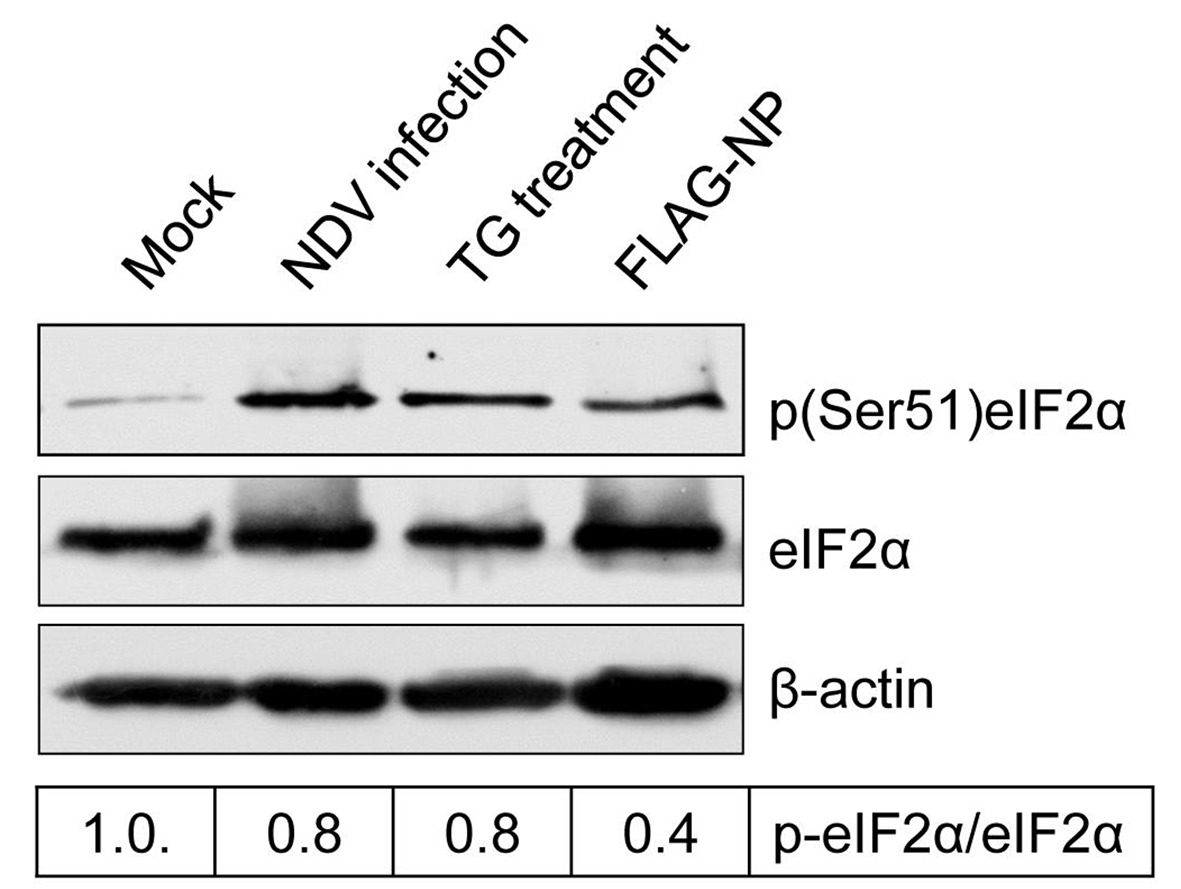

Supplement: S5 Fig — HeLa cells were transfected with FLAG-NP plasmid, infected with NDV at an MOI of 5, or treated with 300 nM Tg for 24 h were harvested for Western blotting analysis of eIF2α, p-eIF2α, and β-actin. The intensities of phospho-eIF2α was determined by densitometry, normalized to total eIF2α. (TIF) [file ppat.1008610.s005.tif]
