# Supplementary material for: Newcastle Disease virus infection activates PI3K/Akt/mTOR and p38 MAPK/Mnk1 pathways to benefit viral mRNA translation via interaction of the viral NP protein and host eIF4E
Source: PLoS Pathog. 2020 Jun 30;16(6):e1008610. doi: 10.1371/journal.ppat.1008610 (PMC7326156; doi:10.1371/journal.ppat.1008610)
Supplement: S1 Table — (DOCX) [file ppat.1008610.s006.docx]

**S1 Table Primers used in this study**

| Mutant | Primer | Application | Sequence (5’-3’) |
| --- | --- | --- | --- |
| pGEX-NP | pGEX-NPF | Cloning and deletion | CGC*GGATCC*ATGTCTTCCGTATTCGACGA |
|  | pGEX-NPR |  | ACGC*GTCGAC*TCAATACCCCCAGTCGGTGT |
| Flag-NP | NPF |  | GA*GAATTC*AATGTCTTCCGTATTCGACGA |
|  | NPR |  | CG*GGATCC*ATACCCCCAGTCGGTGTCATT |
| pCMV-HA-NP | HA-NPF |  | GA*GAATTC*GGATGTCTTCCGTATTCGACGA |
|  | HA-NPR |  | CCG*CTCGAG*TCAATACCCCCAGTCGGTGT |
| Flag-eIF4E | eIF4EF |  | CCC*AAGCTT*ATGGCGACTGTCGAACCG |
|  | eIF4ER |  | CG*GAATTC*GCAACAACAAACCTATTTTTAGTGG |
| Flag-eIF4G | eIF4GF |  | CCC*AAGCTT*ATGTCTGGGGCCCGCACT |
|  | eIF4GR |  | CG*GAATTC*GCGTTGTGGTCAGACTCCTC |
| Flag-NP△C245 | C245F |  | GA*GAATTC*AATGTCTTCCGTATTCGACGA |
|  | C245R |  | CG*GGATCC*GCTCCCACCTGCCGTGTT |
| Flag-NP△N245 | N245F |  | GA*GAATTC*AATGTCCACCTATTACAACTTG |
|  | N245R |  | CG*GGATCC*ATACCCCCAGTCGGTGTCATT |
| Flag-NP△N122 | N122F |  | GA*GAATTC*AATGGAGAGAGCACAGAGATT |
|  | N122R |  | CG*GGATCC*ATACCCCCAGTCGGTGTCATT |
| Flag-NP△C375 | C375F |  | GA*GAATTC*AATGTCTTCCGTATTCGACGA |
|  | C375R |  | CG*GGATCC*ATTGATGCTACTTCCCTGAGC |
| Flag-NP△C380 | C380F |  | GA*GAATTC*AATGTCTTCCGTATTCGACGA |
|  | C380R |  | CG*GGATCC*GGCAGCCATATCCTCATTGAT |
| Flag-NP△C391 | C391F |  | GA*GAATTC*AATGTCTTCCGTATTCGACGA |
|  | C391R |  | CG*GGATCC*GCCCCTCCTTGCTGCTGG |
| Flag-NP△C405 | C405F |  | GA*GAATTC*AATGTCTTCCGTATTCGACGA |
|  | C405R |  | CG*GGATCC*GCTGCCGGTCTCCTCAGA |
| Flag-NP△C440 | C440F |  | GA*GAATTC*AATGTCTTCCGTATTCGACGA |
|  | C440R |  | CG*GGATCC*GGCATCCGGTTGCCCTTG |
| Flag-NP△C464 | C464F |  | GA*GAATTC*AATGTCTTCCGTATTCGACGA |
|  | C464R |  | CG*GGATCC*GGAGTTTGGCGTTTCTCGCA |
| qβ-actin | qβ-actinF | qRT-PCR | GATCTGGCACCACACCTTCT |
|  | qβ-actinR |  | GGGGTGTTGAAGGTCTCAAA |
| qNP | qNPF |  | GAGCGGGAAATCGTGCGTGACAT |
|  | qNPR |  | GGAAGGAAGGTTGGAAGAGAGCC |
| qRL-TK | qRL-TKF |  | AAGGAAACGGATGATAACTG |
|  | qRL-TKR |  | GTCGCCATAAATAAGAAGAG |
| Lenti-NP | Lenti-NPF | Stable expression | CGGCTAGCATGTCTTCCGTATTCGACGA |
|  | Lenti-NPR |  | GCTCTAGATCAATACCCCCAGTCGGTGT |
